# Correction by Focus: Cleft Constructions and the Cross-Linguistic Variation in Phonological Form

**DOI:** 10.3389/fpsyg.2021.648478

**Published:** 2021-11-29

**Authors:** Markus Greif, Stavros Skopeteas

**Affiliations:** ^1^Linguistics Department, Bielefeld University, Bielefeld, Germany; ^2^Linguistics Department, University of Göttingen, Göttingen, Germany

**Keywords:** focus, correction, pitch accent, tonal compression, second occurrence focus, cleft constructions, deaccenting

## Abstract

A challenging issue of cross-linguistic variation is that the same syntactic construction may appear in different arrays of contexts depending on language. For instance, cleft constructions appear with contrastive focus in English, but in a larger array of contexts in French. A part of the cross-linguistic variation may be due to prosodic differences, since prosodic possibilities determine the array of focus structures that can be mapped onto one and the same syntactic configuration. In the present study, we compare languages with flexible nuclear-accent placement (English, German), with languages that do not use this prosodic strategy (French, Mandarin Chinese). In a speech production experiment, we examine the prosodic realization of contrastive focus and identify prosodic reflexes of focus in all languages. The presence of different phonetic reflexes of focus suggests that – anything else being equal – the same syntactic constructions should be possible in the same array of contexts. In an acceptability study with written questionnaires, we examined the felicity of cleft constructions in contexts licensing a focus within the cleft clause. This focus structure is orthogonal to the preferred focus structure of cleft constructions and can appear in cases of second-occurrence foci (in contexts of correction). The obtained judgments reveal a distinction between languages with flexible nuclear-accent placement (English, German) and languages with other types of reflexes of focus (French, Chinese): languages of the former type have an advantage in using cleft constructions with a focus within the cleft clause, which shows that the array of contexts of using clefts in English and German is not a proper subset of the array of contexts applying to the same constructions in French and Chinese. The obtained differences can be explained by the role of prosodic devices and corroborate the view that prosodic reflexes of focus have different semantic-pragmatic import: it is easier to establish a focus structure that is orthogonal to the syntax in a language with flexible nuclear-accent placement (English, German); this does not hold for prosodic correlates of focus that reinforce the articulation of prosodic constituents (French) or the articulation of lexical tones (Chinese).

## Introduction

Discourse notions such as topic and focus are reflected in different grammatical layers, notably in syntax and prosody. The idea that these layers are complementary has been fruitfully used in order to account for the fact that similar syntactic constructions appear in different arrays of contexts depending on language. Vallduví and Engdahl (1996: 497) explain the differences in the use of syntactic movement in Catalan and English in terms of prosodic plasticity. ‘Plastic’ languages, such as English, shift the nuclear stress signaling that the focus is part of the stressed constituent; in ‘non-plastic’ languages, such as Catalan, the nuclear stress appears in a fixed position within the linearization (in case of Catalan, it is the rightmost constituent); syntactic operations are employed such that the focus appears in the position that bears the nuclear stress.^1^ In the same vein, Samek-Lodovici (2005) accounts for the choice of alternative strategies to express focus in English, Italian, and Bantu languages by means of alternating rankings of constraints that sanction deviations from syntactic and prosodic principles. Zubizarreta (1998: 21–22) observes that languages differ with respect to the expression of prosodic prominence of focus. In English, German, and French, clause-initial non-contrastive foci are realized with prosodic prominence followed by deaccenting. In contrast to these languages, Spanish and Italian have a default prosodic prominence on the rightmost prosodic constituent that is not modulated by focus; in order to maintain this prosodic pattern, these languages employ deviations from the canonical word order such that non-contrastive foci surface rightmost in the clause. These approaches share the reasoning that syntactic movement is a last resort, employed for discourse functions that cannot be expressed by prosodic means in the language at issue. The distinction between two classes of languages may be oversimplified, as various instrumental phonetic studies on prosody show (see, e.g., effects of focus on the pitch range of tonal events in Chinese; Xu, 1999). Finally, it is cross-linguistically possible to increase the articulatory effort in order to draw the attention of the hearer to salient parts of the utterance (see effort code in Gussenhoven, 2004: 85–89). However, we know that the exact semantic-pragmatic value of similar prosodic devices can vary between languages (see Vander Klok et al., 2018 for differences in the prosodic means expressing variation in prominence between English and French). Thus, the core question is how different prosodic means of expressing prominence (e.g., nuclear-accent placement in English, pitch range expansion of tonal events in Chinese) can account for the possibility of using the same construction in different contexts depending on language.

Within this line of thought, the present study examines cleft constructions, which are informative for the general question at issue since these constructions are associated with a particular information structure.^2^ In the typical instances of cleft constructions in English, the ‘pivot,’ that is the constituent in the matrix clause, is contrastively focused; this construction asserts that the proposition is true for the pivot to the exclusion of some alternatives that are relevant in discourse (see ‘cleft-focus principle,’ Rochemont, 1986: 133). The ‘cleft clause,’ that is the constituent that surfaces as a relative clause, contains the background information. Example (1) illustrates a context in which the contextual conditions for a felicitous use of the cleft construction are met. In this realization of the cleft, the nuclear stress is aligned with the pivot, as indicated by the small capitals.

(1) A: *Did Mary buy the bicycle?*
B: *No, it’s* JOHN *that bought the bicycle*.

Beyond cleft constructions with a focus in the pivot, as seen in (1), earlier research in English has shown that cleft constructions appear in a variety of contexts such that the focus domain of the utterance is (a part of) the cleft clause (e.g., ‘informative presupposition clefts’ in Prince, 1978; ‘topic-comment clefts’ in Hedberg, 1990, 2013; detailed classification in Delin, 1992; discussion of various classes of examples in Hartmann (2015): 252–270). The information structure of these examples is reflected in prosody: the nuclear accent in informative-presupposition clefts is realized within the cleft clause (see discussion in Delin, 1992, 1995; Hedberg, 2013), while the pivot is not completely deaccented (Hartmann, 2015: 214).

In the present study, we examined a particular type of context that enforces a focus within the cleft clause, namely cases of correction, as introduced in (2). Assume a context containing a cleft construction such that the pivot of the cleft (John) is focused as in (2A). In this context, it is possible to use a cleft construction as in (2B), correcting a part of the utterance in (2A). Correction establishes a relation between an ‘antecedent statement,’ that is available in the discourse, and a ‘corrective statement,’ that is a denial of (a part of) the antecedent statement. The corrective statement contains a replacement that is interpreted as incompatible with the antecedent statement and which is contrastively focused (Steube, 2001; Van Leusen, 2004; Repp, 2010). An important aspect of correction is the *structural parallelism* between the corrective statement and the antecedent statement, which is an instruction to the addressee to identify the relevant statement in discourse (Van Leusen, 2004: 437; Clifton and Frazier, 2016). The effects of structural parallelism are shown in (2): assuming an antecedent statement that contains a cleft construction (for reasons that depend on the contextual conditions of A and are not crucial for our purposes), it is possible to utter a corrective statement as in B, that is structurally parallel to the antecedent claim and involves a contrastive focus within the cleft clause. This configuration deviates from the expectation that the pivot of a cleft construction is the main focus of the utterance.

(2) A: … *It’s* [JOHN]_FOC_ *that bought the car*.
B: *No, it’s* [*John*]_FOC2_ *that bought the* BICYCLE_FOC1_.

The corrective statement in (2B) contains a complex focus structure, involving a primary focus (FOC1) and a secondary focus (FOC2). The primary focus is the focus of the corrective assertion that is expressed by the nuclear accent. The focus on ‘bicycle’ excludes the alternative in the antecedent statement: ‘it’s John that bought the bicycle’ is contrasted to ‘it’s John that bought the car.’ Additionally, this utterance has a second-occurrence focus^3^, FOC_2_, which is inherited from the context utterance. If the cleft construction in (2A) identifies ‘John’ in contrast to further relevant alternatives (e.g., ‘Peter’ or ‘George’), this information is presupposed by the corrective statement in (2B). The second-occurrence focus is expressed by the cleft construction in this case and may have some secondary prosodic prominence (Féry and Ishihara, 2006; Beaver et al., 2007; Howell, 2011; Büring, 2015; Baumann and Ishihara, 2016). The asserted and presupposed information of (2B) can be paraphrased as: ‘it’s John (in contrast to ‘Peter’ or ‘George’) that bought the bicycle (not the car).’

The cleft constructions in (1) and (2) share the interpretation that some contextually relevant alternatives to the pivot are excluded (which applies to further contextual instances of cleft constructions, as shown by Hartmann, 2015: 253). These constructions differ with respect to the partitioning of the utterance in asserted and presupposed information, which is expressed by the nuclear stress placement, as summarized in (3).

(3) Cleft constructions and focus structure
  The pivot of a cleft construction excludes alternatives that are relevant in the context.
  (a) If the nuclear stress falls within the pivot, the exclusion of alternatives is the asserted information (focus).
  (b) If the nuclear stress falls within the cleft clause, the asserted information is in the cleft clause (focus), while the exclusion of alternatives is part of the presupposed information (second-occurrence focus).

The crucial issue is that the variation in the focus structure of cleft constructions requires the possibility of variable nuclear stress placement, as stated in (3). The predictions of (3) are straightforward for languages such as English and German that realize the nuclear stress by means of pitch accents. Our first question is how this contrast can be expressed in languages that do not rely on pitch accents for signaling focus, such as French and Chinese. In order to establish the corresponding prosodic means in these languages, we conducted a cross-linguistic study on speech production (comparing English, German, French, and Chinese), which is reported in Section 2. The results of this study show that reflexes of prosodic prominence appear in all examined languages, but these reflexes are different in nature.

With this background, we examined whether a cleft construction with a focus in the cleft clause is equally felicitous in these languages (Section 3). Judgments of contextual felicity revealed a typological distinction between languages with flexible nuclear-accent placement (English and German) and languages that do not rely on this strategy (French and Chinese). Hence, these findings are in line with the idea that various classes of prosodic events have distinct semantic-pragmatic import: precisely, using cleft constructions with a focus in the cleft clause has an advantage in languages in which nuclear-accent placement unambiguously identifies the intonational nucleus (English and German); see discussion in Section 4.

## Prosodic Reflexes of Focus

### Aims

The present experiment examines whether canonical and cleft constructions can be realized with different prosodic patterns depending on focus in typologically different languages: languages allowing for flexible placement of nuclear accents (English, German), and languages that do not employ this prosodic strategy (French, Chinese).

### Method

#### Participants

Sixteen native speakers of each language participated in this study. They were explained that their participation was voluntary and that the data will be used in anonymized form for research purposes. Written consent (translated into the native language of the participants) was acquired; participants were paid for their contribution to the experiment. Sex was controlled in the samples in order to outbalance the influence of sex on pitch: English (*n* = 16, female = 8, age range = 18–29, average = 22.1; collected in London), German (*n* = 16, female = 8, age range = 19–34, average = 23.4; collected in Bielefeld), French (*n* = 16, female = 8, age range: 18–44 = average 25.9; collected in Lyon), and Chinese (*n* = 16, female = 8, age range = 18–24, average = 20.8; collected in Beijing).

#### Factorial Design

The trials of this study presented short dialogical interactions. The instructor introduced a context, as in (4A). The participant produced a target utterance (4B) containing a corrective statement, whose antecedent was the last sentence of the context.

(4) A: Everyone brought something to the potluck today. Peter brought the bread.
B: No, [Layla]_F_ brought the bread today.

In order to assess the impact of contrastive focus on the prosodic realization of canonical and cleft constructions, we designed an experiment with the factors FOCUS and CONSTRUCTION of the target utterance; see (5). The factor FOCUS refers to the focus domain of the utterance, which depends on the relation of the target utterance to the last sentence of the context, and contains two levels: subject focus and object focus. The factor CONSTRUCTION relates to the syntactic construction of the target utterance: either ‘canonical constructions’ or ‘cleft constructions.’ The target utterance has always the same structure as the antecedent statement, maintaining the structural parallelism of correction as introduced in (2): canonical and cleft constructions in the target utterance always relate to canonical and cleft constructions respectively in the context utterance.

(5) Factorial design of the speech production study
  (a) FOCUS: subject, CONSTRUCTION: canonical
    A: *Everyone brought something to the potluck today. Peter brought the bread.*
    B: *No*, [*Layla*]_F_ *brought the bread today*.
  (b) FOCUS: subject, CONSTRUCTION: cleft
    A: *Everyone brought something to the potluck today. It’s Peter that brought the bread.*
    B: *No, it’s* [*Layla*]_F_ *that brought the bread today*.
  (c) FOCUS: object, CONSTRUCTION: canonical
    A: *Everyone brought something to the potluck today. Layla brought the salad.*
    B: *No, Layla brought the* [*bread*]_F_ *today*.
  (d) FOCUS: object, CONSTRUCTION: cleft
    A: *Everyone brought something to the potluck today. It’s Layla that brought the salad.*
    B: *No, it’s Layla that brought the* [*bread*]_F_ *today.*

#### Material

The experimental conditions were implemented in four items involving different lexicalizations of simple transitive clauses. All lexicalizations had the same syntactic constituents, the same number of syllables and the same word stress pattern (English, German) or tonal structure (Chinese); voiceless obstruents were avoided whenever possible in order to reduce missing values in the *f*_o_ measurements;^4^ see full listing of the items in Supplementary Material, Section 2. The number of items is arguably low. Beyond limitations in developing lexicalizations with the present phonological requirements (same syllabic structure, word stress, tonal structure, avoidance of voiced consonants), the main motivation for this decision is to obtain minimal pairs of prosodic realizations of the same lexicalization and by the same speaker under different treatments. Hence, we created four different lexicalizations in order to obtain four repeated observations with each speaker. The drawback of the limited sample of items is that the findings cannot claim generalizability for the population of possible lexicalizations.

The objects were not final within the utterance, such that tonal events that are associated with object focus do not clash with the final lowering at the right edge of the utterance. Therefore, we used a clause-final temporal adverb in those languages in which the object would otherwise be the rightmost constituent (English and French). These items were recorded in all conditions with all participants, which renders a total of 4 items × 16 participants = 64 tokens per experimental condition (à four conditions: 256 utterances per language). Experimental items were mixed with fillers in a proportion 1 (target): 3 (fillers), whereby a part of the fillers (1:3) were items of a further experiment and the remaining fillers (2:3) were distractors. All trials (targets and fillers) were performed with the same instruction and had the same dialogical structure, as illustrated in (6).

The same types of constructions (canonical constructions vs. cleft constructions) were examined in all languages at issue. German declarative main clauses have a verb-second order, as seen in (6a). Cleft constructions as in (6b) are possible in German but occur less frequently and in restricted contexts compared to English (Dufter, 2009: 168; Fischer, 2009: 90). Narrow focus is usually expressed by prosodic means and/or syntactic movement in German. It is possible to use German cleft constructions with a focus within the cleft clause (Fischer, 2009: 168; Hartmann, 2015: 271), as discussed in Section 1 for English (‘informative presupposition clefts’ in terms of Prince, 1978). Experimental results show that the exhaustive interpretation (i.e., the interpretation that the pivot is the only alternative for which the presupposition of the cleft clause holds true) is not part of the truth-conditional meaning of German clefts (Drenhaus et al., 2011), which differs from English clefts that are exhaustively interpreted (Kiss, 1998: 268; Destruel and De Veaugh-Geiss, 2018).


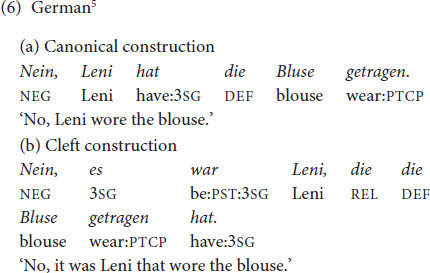


French *c’est* clefts, as in (7), occur in a larger array of contexts than English *it-*clefts. While English clefts are licensed by contrastive focus, French clefts also appear in answers to *wh*- questions (Skopeteas and Fanselow, 2010). Furthermore, French *c’est* clefts with a subject as pivot do not only occur when the subject is in narrow focus, but also whenever the subject is part of a larger focus domain (Lambrecht, 2001; corpus findings in Karssenberg and Lahousse, 2018). While English clefts come with an exhaustive interpretation, this is not necessarily the case for French clefts (Destruel and De Veaugh-Geiss, 2018).


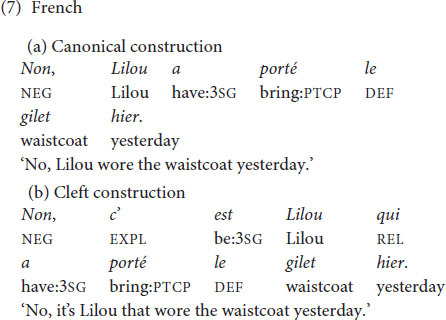


In Chinese, the canonical order with finite verbs is SVO; see (8a) (see discussion in Huang et al., 2009: 199–202). The ‘bare *shi*’ construction in (8b) (with *shi4* preceding the subject) is a cleft construction, typically expressing contrastive focus on the subject. Similarly as with French, the same construction occurs in sentence focus (Cheng, 2008: 255; Paul and Whitman, 2008: 426; Von Prince, 2012: 342; Paul, 2015: 216; see discussion of the tonal properties in Section 2.4).


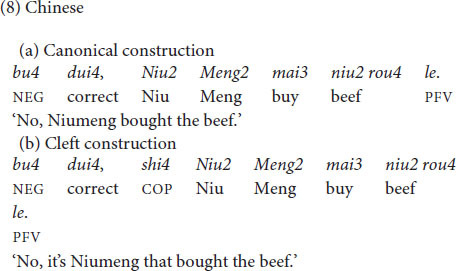


#### Procedure

Recordings took place in quiet rooms in the four places of data collection (London, Bielefeld, Lyon, Beijing). The data was recorded with an Olympus digital recorder (LS-13) with in-built microphones and saved in .wav files at a sampling frequency of 44.1 kHz. The participants were presented with the material in a power point presentation. Each trial was presented in two slides: in a first slide, they read a context-target pair as in (5) and were instructed to look carefully at the dialogue and to memorize the target sentence. In a second slide, only the context was presented, while a native speaker/instructor performed it orally (instructors were advised to perform the context sentences as natural contributions in a dialogue and to avoid a non-expressive style like repeating sentences from a list). The participants were instructed to perform the memorized target utterance in a way that naturally fits to the context (the purpose of this manipulation was to avoid effects of read speech). The participants were allowed to repeat the trial if they thought that their performance was not natural enough (without further guidance by the instructor).

#### Data Analysis

The recordings were processed in praat (Boersma and Weenink, 2020). The data set contained 64 utterances per condition/language; a few tokens had to be removed due to speech disfluencies or errors (two tokens in German and five tokens in Chinese). TextGrid objects were created for the valid data, with intervals corresponding to the syllables of the target utterances. All sound files and TextGrid objects are available at zenodo (Greif and Skopeteas, 2021).

A praat script written by the authors extracted the timing of the onset and the offset of each syllable, as well as the mean *f*_o_ of five equal time bins per syllable. The extracted measurements were processed in *R* (R Core Team, 2020). The *f*_o_ values in Hz were converted into semitones with a reference value of 50 Hz, with the formula *f*_o (semitones)_ = 12(log_2_. *f*_o (Hz)_/50) (Nolan, 2003; Grice et al., 2007; Wang and Xu, 2011).

The *f*_o_ values in semitones were averaged per experimental condition in order to detect the impact of the factors at issue on the *f*_o_ excursion in visualizations. Statistic evaluation was conducted on the non-averaged data.

Linear mixed-effects models were fitted on the (semitone transformed) *f*_o_ measurements in each area of interest (subject or object, see details in Section 2.3) separately (using package *lme4* in R; Bates et al., 2015). We examined *f*_o_ excursions as time series, with the *f*_o_ mean of time bins as dependent variable. The fixed effects were the experimental factors FOCUS (level 0 = object; level 1 = subject) and CONSTRUCTION (level 0 = canonical; level 1 = cleft), and the continuous variable of TIME (levels: 1–5), whose levels refer to the corresponding time bin within the syllable. Including TIME to the model offers the possibility to examine the impact of the fixed effects on the *f*_o_ excursion as a function of time: the interaction effects with TIME reflect the impact of the corresponding fixed factor on the *f*_o_ slope within the area of interest (Barr, 2008). Starting with a random-effects structure with intercepts for PARTICIPANTS and ITEMS as well as by-PARTICIPANTS and by-ITEMS random slopes of FOCUS and CONSTRUCTION, we identified the maximal random-effects structure that converges in all languages for the analyses in a certain area of interest.^6^ Keeping the maximal converging random-effects structure constant (as suggested by Barr et al., 2013), we reduced the fixed-effects structure (FOCUS × CONSTRUCTION × TIME) with a backward-elimination procedure of non-significant effects (performed automatically by the function *step* of the package *lmerTest* in R; Kuznetsova et al., 2017). The fixed effects that were not nested in a higher interaction were additionally tested with Likelihood Ratio Tests (Bates et al., 2015: 35); for the significance of fixed effects that were nested in higher interactions, we can only rely on the *t*-values (ratio of the estimate to its standard error).

### Predictions

The experimental material contains two areas of interest: the *f*_o_ excursion of the subject and *f*_o_ excursion of the object; in languages with stress, either lexical (German, English) or postlexical (French), the area of interest is the corresponding stressed syllable. In the area of the subject, we expect a contrast between nuclear accents (if the subject is focused) and prenuclear accents (if the focus falls on the object); in the area of the object, we expect a contrast between nuclear accents (if the object is focused) and deaccenting (if the focus falls on the subject). In Chinese, we expect that the *f*_o_ excursion of non-focused constituents will be tonally compressed compared to the *f*_o_ excursion of focused constituents (in either area). The type of accent depends on language and will be introduced with the presentation of the results in Section 2.4. In all cases, the expected contrasts imply a difference in the *f*_o_ slope, while the direction of the difference is language-specific (it depends on the prosodic events at issue).

The predictions of this study will be examined by testing for an interaction of the fixed factors with the variable of TIME within the areas of interest (i.e., the syllables in which phonological considerations predict reflexes of focus). Effects of TIME are evidence for a difference in the *f*_o_ slope, reflecting tonal events aligned with the area of interest (Grabe et al., 2007; Isaacs and Watson, 2010). Hence, an interaction FOCUS × TIME or an interaction CONSTRUCTION × TIME indicates that the corresponding fixed factor has an impact on the change of *f*_o_ within the area of interest. Effects that are independent of the time variable, such as a main effect of FOCUS, are evidence for a difference of the *f*_o_ level (see Barr, 2008 concerning the relevance of ‘rate effects’ in time series).

With this background, the major question in cross-linguistic perspective is whether FOCUS × TIME effects appear in all languages. The distinction between plastic (English, German) and non-plastic (French, Chinese) languages predicts that the effects of FOCUS will appear only in the former language type. However, earlier studies have shown that various phonetic reflexes of focus, such as a pitch range expansion or reflexes of demarcation of focused constituents, are found in non-plastic languages as well (see Xu, 1999; Chen and Gussenhoven, 2008 on Chinese and German and D’Imperio, 2010; Delais-Roussarie et al., 2015 on French), which predicts an effect on the *f*_o_ slope in all languages.

An interaction CONSTRUCTION × TIME may appear if certain constructions are associated with prosodic events that are independent of focus. Precisely, cleft constructions differ with respect to prosodic phrasing, such that the cleft clause forms an intonation phrase on its own (Féry, 2013: 699 on French); edge tones that delimit intonation phrases may appear around the boundary between the pivot and the cleft clause.

A threefold interaction FOCUS × CONSTRUCTION × TIME indicates that the effect of FOCUS on the *f*_o_ slope is modulated by CONSTRUCTION. Since cleft constructions with a focus in the cleft clause bear a second-occurrence focus as seen in (2), subject constituents may be not completely deaccented, which predicts a threefold interaction within the area of interest of the subject. In cross-linguistic perspective, effects of second-occurrence focus entail effects of focus. That is, a threefold interaction may appear in a subset of the languages that have a FOCUS × TIME interaction. Our predictions are summarized in (9).

(9) Predicted effects on the *f*_o_ slope
  (a) FOCUS × TIME: focus influences the *f*_o_ slope (language-specific effects).
  (b) CONSTRUCTION × TIME: canonical and clefts constructions differ with respect to p-phrasing.
  (c) FOCUS × CONSTRUCTION × TIME: second-occurrence focus in cleft constructions predicts that the effect of focus on the *f*_o_ slope will be modulated by construction.

### Results

The *f*_o_ excursions in Figure 1 illustrate the basic contrast between early and late foci in British English. Annotations indicate the tonal events that are relevant for our discussion on the prosodic reflexes of focus, assuming the ToBI conventions (Veilleux et al., 2006). When the subject is focused (Figure 1A) it is realized with a bitonal accent L + H*, which stands for a substantial rising pitch movement that reaches a high target within the stressed syllable; this realization is characteristic of contrastive foci in English (Ladd, 2008: 96; Watson et al., 2008; Gotzner, 2015: 130–136). The realization of a subject preceding the focus in Figure 1B also has a rising *f*_o_ excursion, starting from a low target within the stressed syllable and rising toward a high target that may be reached after the stress (L* + H).^7^ The prosodic realization of the objects is different in both figures. When the object is focused, it is realized with a rising contour (Figure 1B), similarly as with the focused subject in Figure 1A. When the object follows the focus, it is deaccented (Figure 1A), which means that it does not contain any significant prosodic events (Ladd, 2008: 231–236) and ends up with a final low target as expected for declaratives, which is phonologically represented by the sequence of a phrase tone (L−) and a boundary tone (L%).

**FIGURE 1:**
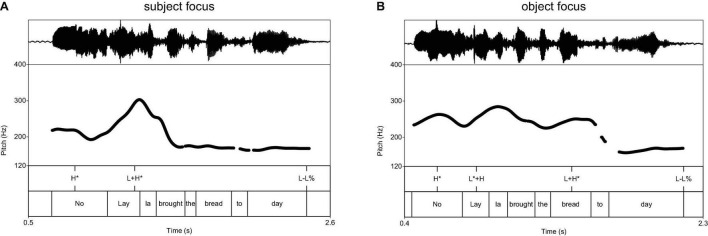
Illustrative examples of **(A)** subject and **(B)** object focus in British English.

The average *f*_o_ excursions of British English (Figure 2)^8^ show a major distinction between early focus (on the subject, blue line) and late focus (on the object, red line), which applies to canonical and cleft constructions. The *f*_o_ rise in the stressed syllable of focused subjects (gray cell) has a greater slope with focus on the subject (blue line) than with focus on the object (red line). The realization of the objects show a rising contour when the object is focused (red line) and is deaccented when the object is given (blue line). These properties apply to canonical and cleft constructions, which means that prosodic marking of focus is not compensated by marking the focus in syntax (see the same effect for Canadian English in Arnhold, 2021).

**FIGURE 2:**
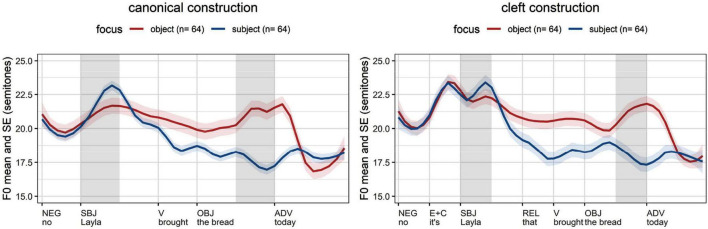
Average *f*_o_ measurements in British English (time normalization based on five equal intervals per syllable; vertical lines: word edges; gray cells: areas of interest, stressed syllable of subject and object).

The German data shows a similar pattern in canonical and cleft constructions (Figure 3). Focused subjects (blue lines) are realized with an *f*_o_ excursion rising up to a H target that is close to the right edge of the stressed syllable, reflecting the fact that German has a bi-tonal accent L + H* for contrastive assertions (Grice et al., 2005: 65, 71; see Alter et al., 2001 on contrast). Non-focused subjects (red lines) optionally have prenuclear accents, reaching an *f*_o_ maximum after the right edge of the stress, which reflects the fact that the H-target of prenuclear accents (L* + H) may follow the stressed syllable (Féry and Kügler, 2008; Baumann and Riester, 2013: 20; Féry, 2017: 154). The impact of focus on object constituents is similar: a rise within the stressed syllable (L + H*) when the object is focused (red lines) viz. deaccented objects with a flat contour when the object is given (blue lines).

**FIGURE 3:**
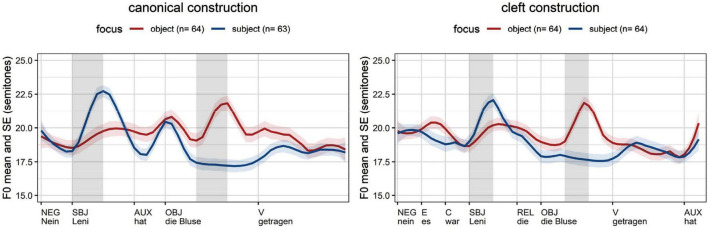
Average *f*_o_ measurements in German (time normalization based on five equal intervals per syllable; vertical lines: word edges; gray cells: areas of interest, stressed syllable of subject and object).

In French, the rightmost full (i.e., non-schwa) syllable is characterized by metrical prominence, which is reflected in lengthening and tonal activity; metrical prominence is assigned postlexically in French, which means that it is not determined by the lexicon (see summary in Post, 2000: 8–9; Féry, 2014). In terms of the French ToBI (Delais-Roussarie et al., 2015), the last syllable of the accentual phrase is associated with a high tonal target (H−), while the last accentual phrase ends up with a low target (L−L%); see Figure 4. French accentual phrases may start with a rise within the initial syllable (German and D’Imperio, 2010; Delais-Roussarie et al., 2015). Since these events are associated with edge syllables, we code them as edge tones associated with the left edge of an accentual phrase (−L + H) (following Féry, 2014). Initial rises are reported to appear more often with contrastively focused constituents (see German and D’Imperio, 2010; Delais-Roussarie et al., 2015), especially in contexts of correction (Vander Klok et al., 2018); however, the function of these events is controversial, since they may be used to draw the attention of the hearer to not focused constituents and there are also empirical studies disputing its correlation with contrastive focus (Cole et al., 2019: 130). The data in Figure 4 illustrate this contrast: focused subjects may be realized with an initial rise (Figure 4A), such that the high target is aligned with the right edge of the first syllable; non-focused subjects are realized with a (lower scaled) high edge tone aligned with the right edge of the accentual phrase (Figure 4B). The initial rise can also appear with focused objects (Figure 4B), while objects are not accented when following the focused subject (Figure 4A).

**FIGURE 4:**
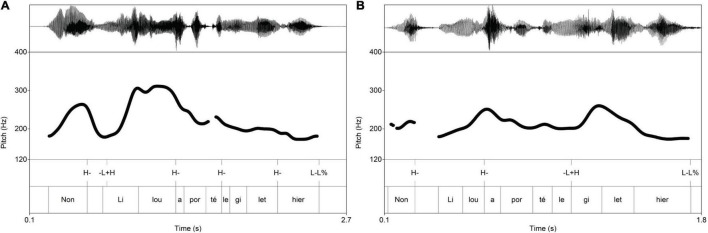
Prosodic realization of **(A)** subject and **(B)** object focus in French.

The averages per experimental condition (Figure 5) confirm that the introduced phenomena depend on information structure. The average *f*_o_ excursion of focused subjects (blue lines) targets an earlier local maximum than the corresponding excursion of non-focused subjects (red lines). Focused objects (red lines) also show an initial rise in contrast to non-focused objects (blue lines). Our data shows that tonal events following the nucleus are not necessarily erased in French (Di Cristo and Jankowski, 1999: 1567; Jun and Fougeron, 2000: 230; Féry, 2014):^9^ prosodic words in the postfocal domain display the same type of *f*_o_ excursion with their focused counterparts – but with a compressed pitch range.

**FIGURE 5:**
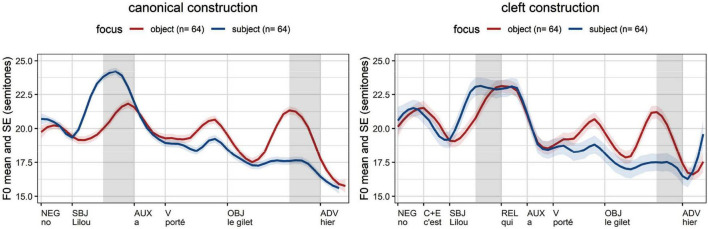
Average *f*_o_ measurements in French (time normalization based on five equal intervals per syllable; vertical lines: word edges; gray cells: areas of interest, stressed syllable of subject and object).

Mandarin Chinese displays a phonological contrast between four lexical tones (T1: high level; T2: rise; T3: fall-rise; T4: fall). The target words in our material contain the simple contour tones T2 and T4 that are comparable since they consist of two tonal targets (i.e., T2: LH, T4: HL). All items have the tonal sequence T2-T2 (rise-rise) for subjects and T2-T4 (rise-fall) for objects; see (8) and Supplementary Material, Section 2.1.4. The choice of T2/T4 was just determined by convenience for the selection of appropriate lexical material and maintained constant across items. Word stress is not applicable to Chinese. Even if some studies report a preference for initial prominence in compounds (Duanmu, 2007: 135, 142), both syllables are areas of interest for our study (see Figure 6), since there is no reason to expect reflexes of focus only in the initial syllable. Focus is reported to be reflected in an expansion of the pitch range of lexical tones, with a greater effect on *f*_o_ maxima than *f*_o_ minima (Xu, 1999: 69; Greif, 2012: 38) as well as by a general increase of the distinctness of tonal targets, which resembles hyperarticulation effects of focus on vowel quality (Chen and Gussenhoven, 2008: 744). This kind of hyperarticulation is also seen in our data: the T2–T2 sequence in the subject is realized with two distinct rising excursions when the subject is focused, but this contour is leveled out into a single rise when the subject is out of focus. A similar contrast applies to the object constituents. The T2–T4 sequence results in a hat contour (LHL), whose peak is reached beyond the offset of the first syllable (Xu and Wang, 2001: 331): this hat contour appears with a reduced pitch range when the object follows the focus, which is evidence for postfocal tonal compression. The asymmetry between prenuclear and postnuclear tonal compression is similar to the asymmetry between prenuclear and postnuclear deaccenting in Germanic languages (Chen, 2010: 520). While the pitch compression is radical in the postnuclear domain, prenuclear tones only slow slight differences in terms of pitch range (see lexical tones of subjects under object focus).

**FIGURE 6:**
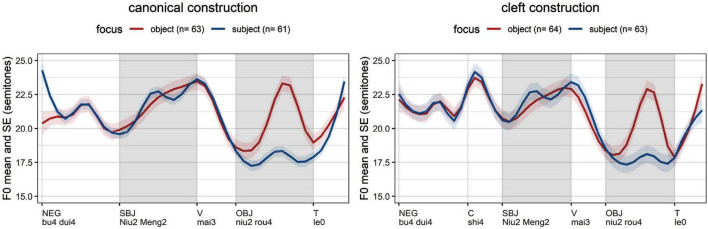
Average *f*_o_ measurements in Chinese (time normalization based on five equal intervals per syllable; vertical lines: word edges; gray cells: areas of interest, subject and object).

Linear mixed-effects models with the factors FOCUS, CONSTRUCTION, and TIME were fitted on the *f*_o_ measurements within the stressed syllables (for objects and subjects separately; see details in 2.2.5). In Chinese, we analyzed the first and the second syllable separately, in order to maintain the same degrees of freedom in all analyses and since we cannot reduce the analysis to a single syllable based on assumptions about word stress.

The maximal random-effects structure that converges in all analyses for subjects contains random intercepts for PARTICIPANTS and ITEMS and a by-PARTICIPANTS random slope of CONSTRUCTION. The models of maximal fit for the *f*_o_ measurements in the stressed syllable of the subject are listed in Table 1. German is the only language with a significant threefold interaction (CONSTRUCTION × FOCUS × TIME), indicating that the effect of FOCUS on the *f*_o_ slope is modulated by CONSTRUCTION, such that the difference between focused and non-focused subjects is greater in canonical clauses (therefore the interaction effect is negative); compare blue and red lines in the area of subjects in Figure 3. In all languages, we obtain a significant FOCUS × TIME interaction, whose direction is language specific: it is positive with rising accents (English, German, Chinese/syllable 1) and negative with falling accents (French). In either case, this effect means that the *f*_o_ change is more rapid when the subject is focused. The models of maximal fit in English and Chinese (syllable 1) contain a negative interaction CONSTRUCTION × TIME, indicating that the *f*_o_ change is slower in cleft than in canonical constructions.

**TABLE 1:** Linear fixed-effects models of best fit on the *f*_o_ measurements (semitones): subject.

Language	Factor	β	SE	*t*	*p* (<)	Likelihood Ratio Test	
						χ^2^	*p* (<)
English	Intercept	19.842	1.494	13.281	0.001		
	CONSTRUCTION (cleft)	1.776	0.197	9.015	0.001		
	FOCUS (subject)	–0.667	0.167	–3.989	0.001		
	TIME	0.409	0.04	10.158	0.001		
	CONSTRUCTION × FOCUS	–0.329	0.131	–2.519	0.05	6.329	0.05
	CONSTRUCTION × TIME	–0.243	0.046	–5.246	0.001	27.218	0.001
	FOCUS × TIME	0.452	0.046	9.769	0.001	91.939	0.001
German	Intercept	18.022	1.226	14.702	0.001		
	CONSTRUCTION (cleft)	0.113	0.254	0.444	–		
	FOCUS (subject)	–1.327	0.21	–6.316	0.001		
	TIME	0.312	0.045	6.994	0.001		
	CONSTRUCTION × FOCUS	0.619	0.296	2.093	0.05		
	CONSTRUCTION × TIME	0.091	0.063	1.449	–		
	FOCUS × TIME	0.912	0.063	14.42	0.001		
	CONSTRUCTION × FOCUS × TIME	–0.302	0.089	–3.388	0.001	11.425	0.001
French	Intercept	19.149	1.182	16.206	0.001		
	CONSTRUCTION (cleft)	1.02	0.259	3.938	0.001		
	FOCUS (subject)	4.996	0.306	16.339	0.001		
	TIME	0.624	0.06	10.406	0.001		
	CONSTRUCTION × FOCUS	–1.87	0.24	–7.787	0.001	59.2	0.001
	FOCUS × TIME	–0.751	0.085	–8.844	0.001	75.843	0.001
Chinese	Intercept	19.107	1.341	14.245	0.001		
(syllable 1)	CONSTRUCTION (cleft)	1.03	0.173	5.968	0.001		
	FOCUS (subject)	–0.729	0.137	–5.304	0.001		
	TIME	0.497	0.036	13.765	0.001		
	CONSTRUCTION × TIME	–0.224	0.041	–5.436	0.001	29.193	0.001
	FOCUS × TIME	0.352	0.041	8.528	0.001	70.603	0.001
Chinese	Intercept	22.623	1.273	17.77	0.001		
(syllable 2)	FOCUS (subject)	0.336	0.146	2.302	0.05		
	TIME	0.245	0.031	7.9	0.001		
	FOCUS × TIME	–0.133	0.044	–3.031	0.01	9.15	0.01

The *f*_o_ measurements in the object constituent reveal similar results in all languages (Table 2). There is a clear interaction effect FOCUS × TIME, which is negative in English, German, and Chinese/syllable 1, since the baseline of object focus is a rise in these languages, while the same syllables in the postfocal domain (subject focus) are rather flat or slightly falling. The corresponding FOCUS × TIME interaction effects are positive in French and in Chinese/syllable 2, in which case the *f*_o_ excursion of the object focus is falling. There is no evidence that the difference between canonical vs. cleft constructions (CONSTRUCTION × TIME) plays a role.

**TABLE 2:** Linear fixed-effects models of best fit on the *f*_o_ measurements (semitones): object.

Language	Factor	β	SE	*t*	*p* (<)	Likelihood-Ratio Test	
						χ^2^	*p* (<)
English	Intercept	19.558	1.445	13.539	0.001		
	FOCUS (subject)	–1.558	0.412	–3.78	0.001		
	TIME	0.413	0.034	12.183	0.001		
	FOCUS × TIME	–0.603	0.05	–12.158	0.001	138.68	0.001
German	Intercept	17.996	1.107	16.259	0.001		
	FOCUS (subject)	0.334	0.286	1.17	–		
	TIME	1.016	0.031	32.392	0.001		
	FOCUS × TIME	–1.003	0.045	–22.074	0.001	406.02	0.001
French	Intercept	22.117	1.292	17.115	0.001		
	CONSTRUCTION (cleft)	–0.569	0.247	–2.31	0.05		
	FOCUS (subject)	–4.666	0.465	–10.039	0.001		
	TIME	–0.536	0.051	–10.531	0.001		
	CONSTRUCTION × FOCUS	0.653	0.205	3.185	0.001	10.095	0.001
	FOCUS × TIME	0.47	0.073	6.485	0.001	41.316	0.001
Chinese	Intercept	17.606	1.396	12.607	0.001		
(syllable 1)	FOCUS (subject)	0.478	0.211	2.266	0.05		
	TIME	0.372	0.032	11.76	0.001		
	FOCUS × TIME	–0.561	0.045	–12.359	0.001	143.51	0.001
Chinese	Intercept	24.411	1.355	18.019	0.001		
(syllable 2)	CONSTRUCTION (cleft)	–0.4	0.14	–2.854	0.05	6.649	0.05
	FOCUS (subject)	–5.343	0.359	–14.897	0.001		
	TIME	–0.841	0.048	–17.407	0.001		
	FOCUS × TIME	0.468	0.07	6.69	0.001	43.902	0.001

### Discussion

The results of the present study reveal that all examined languages show prosodic reflexes of focus, either through the prosodic prominence of the focused constituent or through leveling out the prosodic events of the postfocal domain.

All languages have a significant FOCUS × TIME interaction within the subject area (Table 1), whose properties vary depending on the language-specific tonal events. In German and English, this effect is positive, reflecting the use of rising accents for marking foci in these languages (Grice et al., 2005: 65, 71; Ladd, 2008: 96). A similar effect is found in the first syllable of the subject in Chinese, reflecting a more rapid rise of rising tones (T2) under focus. Our findings are in line with previous results on pitch range expansion of lexical tones under focus, especially applying to the rising tone (T2) (Xu, 1999; Wang and Xu, 2011; Greif, 2012: 75; Ouyang and Kaiser, 2015: 65). In particular, the average contours in Figure 6 show an increase of distinctness between subsequent rises within focus, which is in line with the view that tonal realizations are hyperarticulated under focus (Chen and Gussenhoven, 2008: 744). In French, contrastive focus on the subject frequently induces initial rises in the focused constituent resulting in a falling contour within the last syllable (German and D’Imperio, 2010). Hence, focus has an effect on *f*_o_ excursions in all languages in our sample, as summarized in (10).

(10) Prosodic prominence of focus
  Evidence for prosodic prominence of foci is found in all languages for both subject and object foci and both canonical and cleft constructions. The nature of the obtained effects depends on the specific properties of the languages at issue.
  (a) In English and German the focused constituent bears the nuclear accent, which contains a high peak within the stressed syllable; the effects on the *f*_o_ slope come from the contrast of the nuclear accents with prenuclear accents (area of interest: subject) or with deaccented domains (area of interest: object).
  (b) In French and Chinese, the obtained effects come from phenomena increasing the saliency of prosodic entities: initial rises in French are a general strategy for demarcating prosodic constituents that appear more often with foci; in Chinese, focus is reflected in the hyperarticulation of the tonal targets of phonological events that are independent of focus (lexical tones).

Postnuclear prosodic events are leveled out, which gives rise to a significant FOCUS × TIME interaction in all languages (Table 2). Postnuclear leveling encompasses two types of phenomena, namely deaccenting and tonal compression. In German and English, the postfocal domain is deaccented: the average excursions of postfocal objects reveal a falling contour without any significant prosodic events, sharply contrasting to the corresponding contour of accented constituents. This finding is in line with previous findings in English (Liberman and Pierrehumbert, 1984; Ladd, 2008: 231–236) and German (Féry and Kügler, 2008; Baumann and Riester, 2013: 20; Féry, 2017: 154). The postfocal excursions in French and Chinese have the same prosodic pattern as the corresponding conditions in focus, realized with a reduced pitch range, which is evidence for tonal compression. In French, tonal compression applies to edge tones: the rising contours encompassing prosodic words are visible in focus or out of focus, with a difference in pitch range, which confirms the view that the reflexes of prosodic phrasing on intonation are still visible in the postfocal domain (Di Cristo and Jankowski, 1999: 1567; Jun and Fougeron, 2000: 230; Féry, 2014). In Chinese, tonal compression applies to lexical tones: the hat contour (T2-T4) is realized with reduced pitch range when the object follows the focus, as already reported in instrumental phonetic studies (Xu, 1999: 69; Chen, 2010; Greif, 2012: 82–88, 110–116). This result is not generalizable for all tone languages but confirms the view that Mandarin Chinese belongs to the subclass of tonal languages that have postfocal tonal compression (Xu et al., 2012). Our conclusions are summarized in (11).

(11) Postfocal tonal leveling
  The postfocal domain is prosodically leveled out in all languages:

(a) English and German: the postfocal material is deaccented;
(b) French and Chinese: the available tonal events (edge tones in French, lexical tones in Chinese) are visible after the focus but tonally compressed.

The effects of second-occurrence focus are only confirmed by a significant CONSTRUCTION × FOCUS × TIME interaction in German. This result is in line with previous studies on second-occurrence focus in non-final contexts, in particular Féry and Ishihara (2006) on German. We refrain from any strong statement about a difference between languages with respect to second-occurrence foci: prenuclear accents are optional in general and a prosodic marking of second occurrence focus is not mandatory in these constructions, since it is already expressed through the cleft construction. Nevertheless, the fact that the only language for which we obtained evidence for prosodic reflexes of second-occurrence focus is German is in line with the view that signaling second-occurrence focus entails signaling focus. Languages with a contrast between accent types for the expression of focus are more likely to employ this contrast for second-occurrence foci as well.

Finally, the prosodic devices that can be used for signaling focus are equally used in canonical and cleft constructions. The interaction effects of CONSTRUCTION × TIME in the subject region in English and Chinese are accounted by specific properties of the constructions at issue. In both languages, cleft constructions show a tonal event that is immediately left-adjacent to the first syllable: in English it is a pitch accent aligned with the pronoun *it* (see Figure 2), while in Chinese it is the falling tone (tone 4) on the copula *shi* (see Figure 6). The reflex of these accentual events on the immediately adjacent high target is that the *f*_o_ rise starts later and from a higher pitch level in these constructions, which results into the significant interaction effect in these languages. Hence, this effect relates to language-specific properties of the material and is not informative for a difference between canonical and cleft constructions in terms of the prediction in (9b). An interaction effect of FOCUS × TIME (across constructions) is available in all languages, both in the analyses of subjects (Table 1) as well as in the analyses of objects (Table 2). We conclude from these facts that all languages have the potential to realize different prosodic structures depending on focus with canonical and cleft constructions.

## Contextual Felicity of Syntactic Constructions

### Aims

The aim of the present experiment is to test whether the contextual felicity of cleft constructions with a contrastive focus in the cleft clause depends on the prosodic typology. For this purpose, we collected judgments of the appropriateness of target utterances in certain contexts by means of written questionnaires. The typological distinction between plastic and non-plastic languages (based on the flexibility of nuclear-accent placement) predicts an advantage for languages such as German and English. However, our study on speech production revealed that focus is associated with various reflexes of prosodic prominence in all examined languages (Section 2.4).

### Method

#### Participants

The participants were explained that their participation was voluntary and that the data will be used in anonymized form for research purposes and will be made available through the internet. Participants signed a written consent form (translated into their native language). Participants were paid for their contribution to the experiment studies. This experiment was conducted independently of the experiment on the prosodic reflexes of focus in Section 2 (the participant samples are different). While sex was controlled in the prosodic study, there was no reason to control sex in the study on contextual felicity: English (*n* = 32, female = 14, age range = 18–38, average = 24.3; collected in London), German (*n* = 32, female = 26, age range = 19–32, average = 22.8; collected in Bielefeld), French (*n* = 32, female = 18, age range: 18–46 = average 30.1; collected in Lyon), and Chinese (*n* = 32, female = 28, age range = 18–45, average = 21.9; collected in Beijing).

#### Factorial Design

Participants were presented with a written dialogue containing a context of a speaker A and two alternative target utterances of speaker B (either B_1_ or B_2_) and were instructed to estimate the contextual felicity of the target utterances with respect to the context (see details in Section 3.2.4); see (12).

(12) A: *They auctioned off many things today. Peter sold the bicycle.*
B_1_: *No, John sold the bicycle*.
B_2_: *No, it’s John that sold the bicycle*.

In order to assess the effect of correction in modulating the association of certain constructions with certain focus structures, we designed an experiment with the factors CONSTRUCTION, FOCUS, and CONTEXT; see (13). The context created by speaker A contained an initial sentence that was kept constant across experimental conditions and was used in order to create a richer situation in which alternative focus structures of the final utterance can be accommodated. The target utterances illustrate the two levels of the factor CONSTRUCTION: canonical sentence in B_1_ or cleft construction in B_2_. Their FOCUS (subject or object) depends on their relation to the antecedent statement (last utterance of A). The form of the antecedent statement determines the CONTEXT, being either a canonical or a cleft construction. Cleft constructions are expected to be accommodated in this context by assuming a richer Common Ground: “it’s Peter that sold the car” implies that ‘that somebody sold the car’ is shared knowledge between the interlocutors and the contribution of this utterance to the discourse is that ‘Peter (and not somebody else) did it.’

(13) Factorial design of the contextual felicity study

(a) FOCUS: subject, CONTEXT: canonical
  A: *They auctioned off many things today. Peter sold the bicycle.*
  B_1_: *No*, [*John*]_F_ *sold the bicycle*.
  B_2_: *No*, *it’s* [*John*]_F_ *that sold the bicycle*.
(b) FOCUS: subject, CONTEXT: cleft
  A: *They auctioned off many things today. It’s Peter that sold the car*.
  B_1_: *No,* [*John*]_F_ *sold the bicycle*.
  B_2_: *No, it’s* [*John*]_F_ *that sold the bicycle.*
(c) FOCUS: object, CONTEXT: canonical
  A: *They auctioned off many things today. John sold the car.*
  B_1_: *No, John sold* [*the bicycle*]_*rm F*_.
  B_2_: *No, it’s John that sold* [*the bicycle*]_F_.
(d) FOCUS: object, CONTEXT: cleft
  A: *They auctioned off many things today. It’s John that sold the car*.
  B_1_: *No, John sold* [*the bicycle*]_F_.
  B_2_: *No, it’s John that sold* [*the bicycle*]_F_.

#### Material

The conditions in (13) were implemented in 16 items with different lexicalizations of simple transitive clauses; see item list in Supplementary Material, Section 2.2. The native speakers that created the material were encouraged to create situations that are maximally natural in the languages at issue and contain the target structures – without being necessarily literal translations of the English version. The target utterances contain either canonical or cleft constructions; see discussion of the constructions in Section 2.2.2. The cleft constructions used in the present experiment are illustrated in (14).


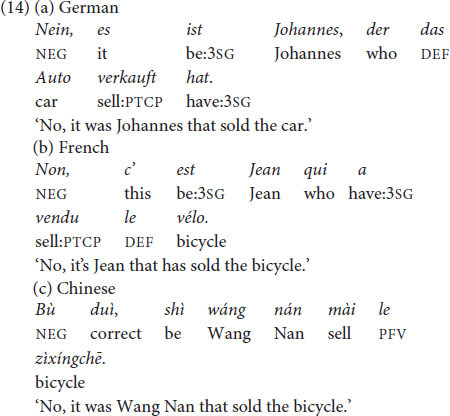


The material was presented in written questionnaires. The background assumption is that participants consider the range of prosodic structures that are active in memory in order to evaluate the felicity of the written utterance in a certain context. Hence, a target utterance may be judged as not felicitous if the participants cannot find an implicit prosodic structure that renders the realization of the utterance congruent with the given context, which may either mean that an appropriate prosodic structure is marginal in language use or that it is not considered sufficient to accommodate the utterance in the given context.

#### Procedure

The participants were presented with a context A and two target utterances B_1_/B_2_ as in (12), whereby each target utterance was accompanied by a scale from 1 to 7 (see Destruel et al., 2019 for a previous study on contextual felicity with a 1–7 scale). The participants were instructed to evaluate the extent that each contribution B_1_/B_2_ was felicitous regarding the context A. The level 1 of the scale stands for ‘the contribution B does not fit to the context A’ and the level 7 for ‘the contribution B fits to the context A.’ The order of presentation of canonical and cleft constructions was randomized in the trials. The reasoning for presenting both utterances in the same trial was motivated by the aim to understand native speakers’ intuitions when considering the paradigmatic alternatives for expressing the same propositional content in certain contexts. We decided to not elicit a single judgment of the comparison between both options since it would not be informative for the felicity of the individual options (two options with the same score could be both felicitous or both non-felicitous).

The material was distributed into four different lists, with each list using each of the 16 items once in a Latin square design. The experimental items of each questionnaire were mixed with fillers at a 1 (targets): 3 (fillers) proportion. Each list was presented to eight participants, which renders (4 lists × 8 participants =) 32 participants (per language). In sum, the dataset of each language contains 16 items × 2 target utterances × 32 participants = 1024 judgments of the contextual felicity of target utterances in context.

#### Data Analysis

The response categories of a Likert scale form an ordinal variable, most importantly because it cannot be warranted that the differences between the numeric values of the 1-to-7 scale reflect equal distances of the estimations of contextual felicity (Bürkner and Vuorre, 2019: 77). We assessed the statistical significance of the examined effects by fitting cumulative link mixed-effects models for ordinal regression (function *clmm* of the package *ordinal* in R; Christensen, 2019). These models estimate the probability of each increase between the levels of the ordinal scale by adding a corresponding intercept to the regression model coefficients (Bürkner and Vuorre, 2019: 79).

The dependent variable of the ordinal regression was the CONTEXTUAL FELICITY, which contains an ordinal scale of ratings (1 to 7). The factors of interest were FOCUS (referring to the focus domain: level 0 = object; level 1 = subject) and CONSTRUCTION (referring to the structure of the target utterance; level 0 = canonical; level 1 = cleft) and CONTEXT (referring to the structure of the last utterance in the context; level 0 = canonical; level 1 = cleft). The random-effects structure contained intercepts for PARTICIPANTS and ITEMS as well as by-PARTICIPANTS and by-ITEMS random slopes of the fixed effects, which converges in all languages; see formula in Supplementary Material, Section 1.2 and see text footnote 6 on the notion of convergence. The maximal fixed-effects structure (FOCUS × CONSTRUCTION × CONTEXT) was reduced with model comparison based on a backward-elimination procedure by means of Likelihood Ratio Tests. The random-effects structure was kept maximal in all compared models (Barr et al., 2013).

### Predictions

The aim of this study is to test whether the contextual felicity of cleft constructions with a focus in the cleft clause is equally felicitous across languages or whether it depends on the prosodic type of the language at issue. The crucial effect for this question is the threefold interaction FOCUS × CONSTRUCTION × CONTEXT, which indicates that the effect of the cleft-focus principle that is reflected in the FOCUS × CONSTRUCTION interaction (subject clefts are felicitous in subject focus) is modulated by CONTEXT, such that the felicity of subject clefts with object focus increases if the antecedent statement is a subject cleft.

The use of cleft constructions with a focus domain in the cleft clause is the configuration introduced in (2), which requires an expression of the focus by prosodic means that deviates from the preferred focus structure of a cleft construction. Hence, the cross-linguistic question is whether this threefold interaction will appear in all languages or in a phonologically determined subtype of languages (see discussion in Section 1). The null hypothesis is that this configuration will be possible in all languages of our sample, since they have been shown to have prosodic reflexes of focus (see Section 2.4). However, we have seen that the observed effects come from different types of phenomena: English and German use certain pitch accents that unambiguously determine the intonational nucleus of the utterance (nuclear accents) and correspondingly the focus placement, while the effects in French and Chinese are general indicators of prosodic prominence (not reserved for focus), which maximize the demarcation of prosodic constituents (edge tones in French) or tonal targets (lexical tones in Chinese). If this difference is relevant for expressing different focus domains with one and the same syntactic construction, then we should obtain a three-way interaction in German/English and not so in French and Chinese. The predictions of our study are summarized in (15).

(15) Predicted interaction effects on CONTEXTUAL FELICITY
  (a) CONSTRUCTION × CONTEXT: an advantage for using the same construction in the target utterance (CONSTRUCTION) and the antecedent utterance (CONTEXT) is predicted by syntactic priming in general and additionally by the preference for structural parallelism between corrective statements and their antecedents (Van Leusen, 2004: 437; Clifton and Frazier, 2016; see Section 1).
  (b) FOCUS × CONSTRUCTION: an advantage for subject clefts in subject focus is predicted by the cleft-focus principle (Rochemont, 1986: 133; see Section 1).
  (c) FOCUS × CONSTRUCTION × CONTEXT: the FOCUS × CONSTRUCTION interaction is modulated by CONTEXT; in particular a second-occurrence focus is expected to result in an advantage for a focus within a cleft clause if the antecedent statement has the same syntactic construction. The three-way interaction is expected to appear only in English/German if reflexes of second-occurrence focus only apply to languages with unambiguous cues of the intonational nucleus, or in all languages otherwise.

### Results

The averaged judgments reveal a major difference between plastic (English and German) and non-plastic (French and Chinese) languages (see Figure 7). The canonical sentences in English and German are almost equally felicitous in subject and object focus, while subject cleft constructions are more felicitous with subject focus. The interesting result is the contextual felicity of subject clefts in an object focus context. In this case, we observe a difference depending on the structure of the context utterance: if this utterance is a cleft (gray dots), then the contextual felicity of the cleft construction increases. Finally, cleft constructions in German generally obtain lower judgments than the same constructions in English. French and Chinese differ. Canonical target utterances (solid lines) show an effect of FOCUS, such that the contextual felicity decreases with subject focus. The judgments of cleft constructions (dashed lines) show the mirror image, rendering a disordinal interaction: subject clefts in French and Chinese are highly felicitous with subject foci and not so with object foci. Crucially, the context utterance has a marginal role in these languages. In the non-canonical constructions, we observe a slight advantage of contextual felicity with object focus, when the same construction is presented in the context: see difference between gray and black dots in object focus with non-canonical constructions (dashed lines).

**FIGURE 7:**
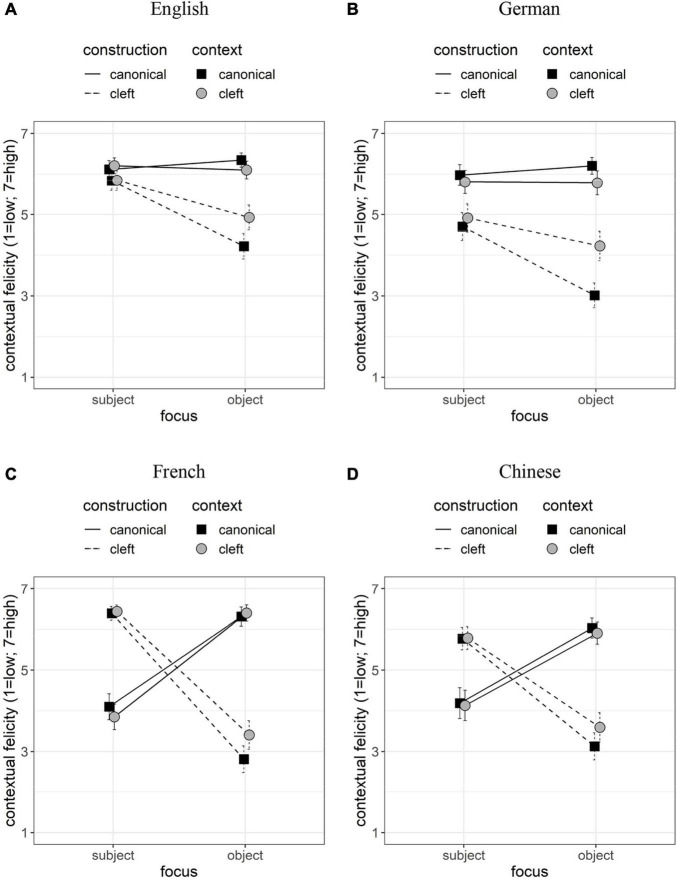
(A–D) Contextual felicity (Y-bars: confidence intervals with probability 0.95).

Cumulative link mixed-effects models for ordinal regression were fitted on the ratings of contextual felicity of each language separately (see Section 3.2.5). The estimates assessing the effect of each increase between the levels of the Likert scale (1 to 7) are listed in Supplementary Material, Section 4. These intercept values are added to the model coefficients rendering the logit of the probability that the outcome exceeds a certain threshold. The relevant information for our data is the general tendency that is captured by the average estimates in (16): the estimates of edge values have greater differences (2|3 minus 1|2 renders 1.6 on average; 6|7 minus 5|6 renders 1.4) than estimates of middle values (all further average differences are below 1). Hence, the estimates reveal that the levels of the Likert scale are not equidistant.

(16) Average threshold intercepts of the increases in the ordinal-scale ratings (1 to 7)
  1|2, average estimate: −6.177
  2|3, average estimate: −4.767
  3|4, average estimate: −3.834
  4|5, average estimate: −3.136
  5|6, average estimate: −2.161
  6|7, average estimate: −0.510

The coefficients of the fixed factors (Table 3) show that all models contain a significant effect of CONSTRUCTION × FOCUS, indicating that cleft constructions with a subject pivot reach a better fit (compared to the canonical constructions) if the subject is focused. While the threefold interaction (CONSTRUCTION × FOCUS × CONTEXT) is significant in English and German, it is not so in French and Chinese. The negative interaction effect indicates that the effect of the cleft-focus principle (CONSTRUCTION × FOCUS) is modulated by CONSTRUCTION, such that contextual felicity increases in the baseline of the FOCUS factor (object focus) with subject clefts in the target utterance (CONSTRUCTION) and the context (CONTEXT). In English, German, Chinese, we obtained a positive interaction CONSTRUCTION × CONTEXT, which means an advantage for using the same construction in the target as in the immediate context. The model of maximal fit in French contains a negative FOCUS × CONTEXT interaction, reflecting the fact that subject clefts obtain higher ratings than canonical constructions under subject focus but lower ratings under object focus (see Figure 7C). The main effect of CONSTRUCTION is negative in all languages, since canonical constructions achieve higher scores than cleft constructions across contexts. The main effect of FOCUS is also negative, reflecting a subject vs. non-subject asymmetry in focus. The factor CONTEXT has a significant negative effect in German (cleft constructions in the context utterance are judged to be less felicitous) and a significant positive effect in French (cleft constructions in the context utterance are judged to be more felicitous).

**TABLE 3:** Cumulative link models on the ordinal scale of contextual felicity.

Language	Factor	β	SE	*z*	*p* <	Log-Likelihood Test	
						χ^2^	*p* <
English	CONSTRUCTION (cleft)	–3.817	0.402	–9.498	0.001		
	FOCUS (subject)	–0.269	0.342	–0.788	–		
	CONTEXT (cleft)	–0.478	0.326	–1.466	–		
	CONSTRUCTION × FOCUS	2.767	0.388	7.127	0.001		
	CONSTRUCTION × CONTEXT	1.487	0.37	4.017	0.001		
	FOCUS × CONTEXT	0.736	0.393	1.872	–		
	CONSTRUCTION × FOCUS × CONTEXT	–1.592	0.522	–3.048	0.01	9.351	0.01
German	CONSTRUCTION (cleft)	–4.288	0.377	–11.375	0.001		
	FOCUS (subject)	–0.351	0.339	–1.035	–		
	CONTEXT (cleft)	–0.593	0.271	–2.187	0.05		
	CONSTRUCTION × FOCUS	2.3	0.361	6.376	0.001		
	CONSTRUCTION × CONTEXT	1.871	0.356	5.26	0.001		
	FOCUS × CONTEXT	0.353	0.371	0.952	–		
	CONSTRUCTION × FOCUS × CONTEXT	–1.288	0.494	–2.605	0.01	6.813	0.01
French	CONSTRUCTION (cleft)	–5.059	0.308	–16.424	0.001		
	FOCUS (subject)	–3.579	0.266	–13.434	0.001		
	CONTEXT (cleft)	0.522	0.184	2.839	0.01		
	CONSTRUCTION × FOCUS	8.65	0.377	22.971	0.001	798.87	0.001
	FOCUS × CONTEXT	–0.629	0.247	–2.547	0.05	6.506	0.05
Chinese	CONSTRUCTION (cleft)	–3.571	0.367	–9.732	0.001		
	FOCUS (subject)	–2.378	0.221	–10.744	0.001		
	CONTEXT (cleft)	–0.197	0.181	–1.091	–		
	CONSTRUCTION × FOCUS	5.358	0.293	18.311	0.001	410.88	0.001
	CONSTRUCTION × CONTEXT	0.604	0.242	2.494	0.05	6.25	0.05

### Discussion

Our findings show a major contrast between English and German on the one side and French and Chinese on the other. The major issue is the difference between languages: the felicity of the same constructions is judged differently under identical treatments depending on language. It is not the case that constructions with low scores are impossible. For instance, canonical sentences with a subject focus are possible albeit not the preferred option in French (Destruel, 2013: 162, Destruel, 2016: 310); cleft constructions with a focus in the embedded clause are possible in French (Dufter, 2009: 105, 114) and Chinese (Yan and Calhoun, 2019).

A first difference between languages relates to the felicity conditions of canonical sentences. The results for English and German indicate that canonical sentences are contextually unrestricted, as expected for languages with flexible placement of the nuclear accent. This result is in line with statements about the optionality of cleft constructions in English: cleft constructions are optionally used to express focus since the focus can be unambiguously identified through the phonological form (Kiss, 1998: 268). Crucially, the judgments differ for French and Chinese, in which case the contextual felicity of canonical sentences radically drops if the subject is focused. This result confirms intuitions about a constraint against focus on preverbal subjects in French (Lambrecht, 2001: 492; Hamlaoui, 2007), which is accounted for by the general preference of French for aligning the focus with the right edge of the intonation phrase (Féry, 2013: 698). Studies on speech production show that subjects are mostly focused through cleft constructions (Destruel, 2013: 162; Destruel, 2016: 310). Similar effects are reported for Chinese: canonical SVO sentences are typically mapped on a Topic-Comment articulation, which has specificity effects on the interpretation of preverbal subjects (Huang et al., 2009: 200). These effects are part of a cross-linguistic preference to map subjects on topics, which results to a subject vs. non-subject asymmetry in marking focus, such that subject focus often appears with additional marking (Lambrecht, 2001: 490; Hartmann and Zimmermann, 2007; Zerbian, 2007: 336; Destruel, 2016: 304). In our findings, the preference against a subject focus in canonical constructions depends on language; see (17).

(17) Contextual conditions for canonical constructions
  In English and German, canonical constructions are judged as equally felicitous in subject and object focus contexts. In French and Chinese, canonical constructions are judged to be less felicitous in subject focus contexts.

The felicity of the subject clefts under subject focus confirms the association of the pivot of cleft constructions with contrastive focus. The only language in which subject clefts are judged differently from canonical sentences in subject focus is German; this finding is in line with the fact that cleft constructions in German are less frequent in corpora than the same constructions in Romance languages (Dufter, 2009: 90). The crucial finding of this study is the significant FOCUS × CONTEXT interaction in English and German – in contrast to French and Chinese. This result confirms the expectations concerning the flexibility in nuclear-accent placement. If the placement of the nuclear accent is flexible, as it is assumed for English and German, the use of cleft constructions with a later nuclear accent has an advantage in contextual felicity (in appropriate contexts); see (18). The question is how this finding combines with the results of the speech production study (see general discussion in Section 4).

(18) Contextual conditions for cleft constructions
  Across languages, the felicity of subject clefts increases if the pivot is focused. In English and German, but not in French and Chinese, the contextual felicity of cleft constructions with a focus in the cleft clause increases when the context motivates the use of the cleft construction (in our manipulation by the structural parallelism of a corrective statement to an antecedent statement).

A final note is due concerning the main effects of the fixed factors. The negative effect of CONSTRUCTION reflects the fact that cleft constructions are contextually restricted in comparison to canonical constructions. The negative effect of FOCUS reflects the subject vs. non-subject asymmetry in focus: the preferred option in discourse is a focus on objects or further verbal complements, while focus on subjects is the least preferred case (Lambrecht, 2001: 490; Hartmann and Zimmermann, 2007; Zerbian, 2007: 336; Destruel, 2016: 304). The effect of CONTEXT is negative in German and positive in French. This effect relates to the felicity of the context utterance (independent of the corresponding target). In a language such as German, in which cleft constructions are highly marked and rare in discourse, the presence of a cleft construction without an obvious contextual trigger in the context, is judged to be suboptimal. The opposite effect appears in French, a language in which subject clefts are very frequent in discourse and may appear without requiring a focus on the subject.

## General Discussion and Conclusion

The presented studies examined hypotheses concerning the interaction of prosody and syntax in the expression of information structure. A reasonable hypothesis is the idea that the reflexes of focus on different layers of grammar are complementary: the syntactic expression of discourse notions is motivated if they are not expressed by prosody. Several versions of this complementarity have been used to explain cross-linguistic differences in the use of cleft constructions and other syntactic operations (Vallduví and Engdahl, 1996: 497; Zubizarreta, 1998: 89; Samek-Lodovici, 2005; discussion in Section 1).

We compared the use of cleft constructions in contexts licensing contrastive focus in four languages with different prosodic properties: two languages allowing for flexible nuclear-accent placement (English, German), a language that relies on prosodic phrasing (French) and a language with lexical tones (Mandarin Chinese). A speech production study has shown that all languages show prosodic reflexes of focus: the contrast between nuclear and prenuclear accents in English and German, initial rises demarcating prosodic constituents in French, increase of the distinctness of tonal targets in Chinese; see (10). The postfocal domain is also affected by effects of prosodic leveling in all languages, deaccenting in English and German and compression of edge and lexical tones in French and Chinese; see (11).

In a study on contextual felicity, we examined whether canonical and cleft constructions can be used in contexts licensing a focus on the subject and on the object. This study reveals a typological distinction between two classes of languages. Canonical constructions are contextually unrestricted in English and German, but less felicitous with subject focus in French and Chinese; see (17). Cleft constructions are more felicitous with a nucleus in the cleft clause, if the context motivates the use of the cleft as a corrective statement that relates to a cleft construction within the antecedent statement: crucially, this effect was statistically confirmed for English and German, but not for French and Chinese; see (18). This result has repercussions for the interpretation of the data collected in speech production. Our basic assumption regarding the contextual felicity judgments is that the participants evaluate a syntactic construction as felicitous in the presented context if they may recall a prosodic structure that fits to this context. Low scores of contextual felicity indicate that this structure is not active in memory, which may mean that this construction is marginal in language use. We conclude from the findings of this study that the French and Chinese data with focus on the cleft clause that we collected in speech production are marginal in language use.

Previous studies have shown that cleft constructions occur in a wider array of contexts in French and Chinese than in English and German. Cleft constructions require a contrastive context in English, while in French the same constructions also appear in non-contrastive contexts, such as answers to *wh*- questions (Skopeteas and Fanselow, 2010). In the same vein but based on acceptability and response time data, Destruel and De Veaugh-Geiss (2018) conclude that an exhaustive inference is part of the default interpretation of English clefts, which does not hold true in French. Beyond narrow focus on the subject, cleft constructions also appear in contexts in which the subject is part of a broader focus domain, such as sentence focus in French (Lambrecht, 2001; Karssenberg and Lahousse, 2018) and Chinese (Paul and Whitman, 2008: 426; Von Prince, 2012: 342), which does not apply to English and German (Dufter, 2009: 114). These comparisons may lead to the conclusion that cleft constructions are semantically bleached in French and Chinese and not so in English and German, such that they appear in a wider array of contexts in the latter type of languages than in the former.

However, our findings identified a type of context (focus within the cleft clause) in which cleft constructions have an advantage only in English and German. Hence, the array of contexts of English and German clefts is not a proper subset of the array of contexts of French and Chinese clefts, which is against the prediction of bleaching. A view from prosodic typology is relevant for understanding this difference, since the crucial context has exactly the property of requiring a prosodic marking of focus. A prosodic account also explains the occurrence of clefts in a wider array of focus types in French and Chinese: if cleft formation is the only means to focus a subject, as in these languages, then it follows that it will appear in any context involving subject focus, without contextual restrictions such as contrastivity or exhaustivity.

The question is how this typological distinction relates to the prosodic findings of the speech production study, which has shown that all languages have some prosodic reflexes of information structure. Significant prosodic effects of focus were found in all four languages in line with earlier findings; see Vander Klok et al. (2018) on reflexes of different types of focus in French, Greif (2012) and Ouyang and Kaiser (2015) for the impact of contrastive focus in Chinese as well as Yan and Calhoun (2019) for effects of prosodic prominence in Chinese on interpretation (invoking alternatives). Moreover, our study shows that these prosodic effects equally appear in the constructions at issue (canonical and cleft constructions).

The crucial observation is that the prosodic reflexes found in these languages come from different classes of phenomena, as outlined in (10). In English and German, the focus determines the placement of the nuclear stress, which is reflected on the contrast between nuclear and prenuclear accents (area of interest: subject) or the contrast between nuclear accents and deaccenting (area of interest: object). On the other hand, focus is reflected on events that reinforce the articulation of prosodic constituents in French (initial rise) or the articulation of lexical tones in Chinese (distinctness of tonal targets). These classes of phenomena have distinct semantic-pragmatic import, such that only the first class of phenomena is an unambiguous indicator of the focus structure of the utterance. While nuclear-accent placement is directly determined by the focus structure, effects on the articulation of prosodic constituents or lexical tones may be employed in order to draw the attention of the hearer to a certain partition of the utterance without being unambiguously associated with a focus structure. The cross-linguistic differences in the flexibility of using canonical and cleft constructions in various contexts is straightforwardly accounted for by this distinction: our findings show that determining a layer of focus structure that is independent from syntax has an advantage in languages of the former type.

## Data Availability Statement

The original contributions presented in the study are included in the article/Supplementary Material, further inquiries can be directed to the corresponding author.

## Ethics Statement

Ethical review and approval was not required for the study on human participants in accordance with the local legislation and institutional requirements. The patients/participants provided their written informed consent to participate in this study.

## Author Contributions

MG and SS designed the experiments, analyzed the data, and prepared a first draft together. MG supervised the data collection and data processing in the individual languages. SS prepared the final manuscript. Both authors reviewed the manuscript and approved the final version to be published.

## Conflict of Interest

The authors declare that the research was conducted in the absence of any commercial or financial relationships that could be construed as a potential conflict of interest.

## Publisher’s Note

All claims expressed in this article are solely those of the authors and do not necessarily represent those of their affiliated organizations, or those of the publisher, the editors and the reviewers. Any product that may be evaluated in this article, or claim that may be made by its manufacturer, is not guaranteed or endorsed by the publisher.

## Footnotes

1 The concept ‘nuclear stress’ refers to the maximal prosodic prominence within an intonation phrase and is underspecified with respect to the phonetic reflexes of prominence. The concept ‘nuclear accent’ refers to the pitch accent that realizes the nuclear stress (in languages expressing prosodic prominence by means of pitch accents).

2 Cleft constructions are analyzed as the result of syntactic movement (see Kiss, 1998), but the exact syntactic analysis of cleft constructions is not crucial for the argumentation of the present article. The crucial issue is that cleft constructions convey the same propositional content as the corresponding canonical constructions with a different information structure.

3 Second-occurrence foci refer to expressions that contain the focus domain of a relevant statement within the background partition of the utterance (see Krifka, 1997; Rooth, 1996, 2009; Büring, 2015).

4 For the fundamental frequency we use the notation *f*_o_, whereby “o” stands for oscillation (Titze et al., 2015).

6 A model fails to “converge” if the procedure estimating the model parameters does not find a solution within the defined number of iterations. A common treatment of this problem is to simplify the random-effects structure, since the likelihood of convergence failure increases with the complexity of model parameters (Barr et al., 2013: 261).

7 Prenuclear accents may make various contributions to the meaning of sentences (Baumann and Winter, 2018; Cole et al., 2019), but they are not necessarily associated with information structure since they may just reflect the rhythmical organization of the utterance (Baumann et al., 2021). The relevant aspect for our data is that prenuclear accents may be particularly weak (in terms of *f*_o_ scaling) if they precede narrow foci as in our data (Calhoun, 2010: 28).

8 Abbreviations for Figures: ADV, adverb; AUX: auxiliary; C, copula; E, expletive; NEG, negation; OBJ, object; T, tense; REL, relative; SBJ, subject; V, lexical verb.

9 Di Cristo and Jankowski (1999: 1567) report that postnuclear pitch variation is reduced, but not eliminated, Jun and Fougeron (2000: 230) conclude that postnuclear domains are deaccented but not dephrased, Féry (2014) argues that only prosodic phrases but not prosodic words are deaccented in French.
