# Supplementary material for: Correction by Focus: Cleft Constructions and the Cross-Linguistic Variation in Phonological Form
Source: Front Psychol. 2021 Nov 29;12:648478. doi: 10.3389/fpsyg.2021.648478 (PMC8666418; doi:10.3389/fpsyg.2021.648478)
Supplement: Supplementary file 1 [file Data_Sheet_1.pdf]

Supplementary Material

Contents

1 Data processing 1
1.1 R and R-package versions 1
1.2 Statistic procedures 2
2 Item lists 3
2.1 Speech production study 3
2.2 Contextual felicity study 5
3 R-code 9
3.1 Speech production study 9
3.2 Contextual felicity study 14
4 Ordinal regression: threshold intercepts 18

1 Data processing

1.1 R and R-package versions

```
> version[['version.string']]
[1] "R version 4.0.3 (2020-10-10)"
> packageVersion("lme4")
[1] ‘1.1.26’
> packageVersion("lmerTest")
[1] ‘3.1.3’
> packageVersion("ordinal")
[1] ‘2019.12.10’
```

1.2 Statistic procedures

Linear mixed-effects model fitted to the f_0 measurements of the speech production experiment:

Dependent variable: f_0 mean of a time bin in semitones

Fixed factors and levels: CONSTRUCTION: canonical|cleft

FOCUS: object|subject

TIME: numeric variable from 1 to 5, corresponding to five equal time bins of the syllable

Model: Starting from a random-effects structure with intercepts of PARTICIPANTS and ITEMS and their slopes with CONSTRUCTION and FOCUS, we identified the maximal random-effects structure that converges with the measurements of the area of interest (subject or object) in all languages:

(a) Analysis of subject measurements

```
lmer(fo_sem ~ construction*focus*time +
          (1 + construction | participant) +
          (1 | item),
          data=dta, REML=FALSE)
```

(b) Analysis of object measurements

```
lmer(fo_sem ~ construction*focus*time +
          (1 + focus + construction | participant) +
          (1 | item),
          data=dta, REML=FALSE)
```

A cumulative link mixed-effects model fitted to the contextual felicity judgments

Dependent variable: 1 to 7 ratings (as ordinal data)

Fixed factors and levels: FOCUS: object|subject

CONSTRUCTION: canonical|cleft

CONTEXT: canonical|cleft

Model: The random-effects structure used for the analyses of the ratings is the following (this is the maximal structure that converges in all languages):

```
clmm(rating ~ construction * focus * context +
          (1+ construction + focus + context | participant) +
```

```
(1+ construction + focus + context | item),
data=dataset)
```

2 Item lists

2.1 Speech production study

Abbreviations: ADV: adverb; C: copula; E: expletive; NEG: negation; OBJ: object; T: tense; REL: relative; SBJ: subject; V: lexical verb.

2.1.1 English

item	target	NEG	E+C	SBJ*		REL	V	OBJ		ADV	
1	plain	*No*		*Lay*	*la*		*brought*	*the*	*bread*	*to*	*day*
	cleft	*No*	*it's*	*Lay*	*la*	*that*	*brought*	*the*	*bread*	*to*	*day*
2	plain	*No*		*Mi*	*lo*		*helped*	*the*	*groom*	*to*	*day*
	cleft	*No*	*it's*	*Mi*	*lo*	*that*	*helped*	*the*	*groom*	*to*	*day*
3	plain	*No*		*Mi*	*ra*		*took*	*the*	*bream*	*to*	*day*
	cleft	*No*	*it's*	*Mi*	*ra*	*that*	*took*	*the*	*bream*	*to*	*day*
4	plain	*No*		*Ni*	*na*		*ate*	*the*	*brain*	*to*	*day*
	cleft	*No*	*it's*	*Ni*	*na*	*that*	*ate*	*the*	*brain*	*to*	*day*

2.1.2 German

item	target	NEG	E	C	SBJ		AUX	REL	OBJ			V			AUX
1	plain	*Nein*			*Le*	*ni*	*hat*		*die*	*Blu*	*se*	*ge*	*tra*	*gen*	
	cleft	*Nein*	*es*	*war*	*Le*	*ni*		*die*	*die*	*Blu*	*se*	*ge*	*tra*	*gen*	*hat*
		'No, (it's) Leni (that) wore the blouse.'													
2	plain	*Nein*			*Ne*	*le*	*hat*		*die*	*Wän*	*de*	*ge*	*stri*	*chen*	
	cleft	*Nein*	*es*	*war*	*Ne*	*le*		*die*	*die*	*Wän*	*de*	*ge*	*stri*	*chen*	*hat*
		'No, (it's) Nele (that) painted the walls.'													
3	plain	*Nein*			*La*	*ra*	*hat*		*die*	*Bä*	*ren*	*ge*	*füt*	*tert*	
	cleft	*Nein*	*es*	*war*	*La*	*ra*		*die*	*die*	*Bä*	*ren*	*ge*	*füt*	*tert*	*hat*
		'No, (it's) Lara (that) fed the bears.'													
4	plain	*Nein*			*Le*	*na*	*hat*		*die*	*Glä*	*ser*	*zer*	*schla*	*gen*	
	cleft	*Nein*	*es*	*war*	*Le*	*na*		*die*	*die*	*Glä*	*ser*	*zer*	*schla*	*gen*	*hat*
		'No, (it's) Lena (that) hit the glasses.'													

2.1.3 French

item	target	NEG	C+E	SBJ		REL	AUX	V		OBJ			ADV
1	plain	*Non*		*Li*	*lou*		*a*	*por*	*té*	*le*	*gi*	*let*	*hier*
	cleft	*Non*	*c'est*	*Li*	*lou*	*qui*	*a*	*por*	*té*	*le*	*gi*	*let*	*hier*

		'No, (it's) Lilou (that) wore the waistcoat yesterday.'											
2	plain	*Non*		*Nel*	*ly*		*a*	*soi*	*gné*	*la*	*brû*	*lure*	*hier*
	cleft	*Non*	*c'est*	*Nel*	*ly*	*qui*	*a*	*soi*	*gné*	*la*	*brû*	*lure*	*hier*
		'No, (it's) Nelly (that) treated the burn yesterday.'											
3	plain	*Non*		*Li*	*ly*		*a*	*nour*	*ri*	*le*	*bi*	*son*	*hier*
	cleft	*Non*	*c'est*	*Li*	*ly*	*qui*	*a*	*nour*	*ri*	*le*	*bi*	*son*	*hier*
		'No, (it's) Lily (that) fed the buffalo yesterday.'											
4	plain	*Non*		*Lo*	*la*		*a*	*dé*	*truit*	*le*	*man*	*drin*	*hier*
	cleft	*Non*	*c'est*	*Lo*	*la*	*qui*	*a*	*dé*	*truit*	*le*	*man*	*drin*	*hier*
		'No, (it's) Lola (that) destroyed the chuck yesterday.'											

2.1.4 Chinese

item	target	NEG		C	SBJ		V	OBJ		T
1		不	对,	(是)	牛	萌	买	牛	肉	了
	plain	bu4	dui4		Niu2	Meng2	mai3	niu2	rou4	le
	cleft	bu4	dui4	shi4	Niu2	Meng2	mai3	niu2	rou4	le
		'No, (it's) Niu Meng (that) bought the beef.'								
2		不	对,	(是)	毛	玲	问	门	卫	了
	plain	bu4	dui4		Mao2	Ling2	wen4	men2	wei4	le
	cleft	bu4	dui4	shi4	Mao2	Ling2	wen4	men2	wei4	le
		'No, (it's) Mao Ling (that) asked the guard.'								
3		不	对,	(是)	罗	兰	写	邮	件	了
	plain	Bu4	dui4		Luo2	Lan2	xie3	you2	jian4	le
	cleft	Bu4	dui4	shi4	Luo2	Lan2	xie3	you2	jian4	le
		'No, (it's) Luo Lan (that) wrote the letter.'								
4		不	对,	(是)	刘	林	做	凉	面	了
	plain	Bu4	dui4		Liu2	Lin2	zuo4	liang2	mian4	le
	cleft	Bu4	dui4	shi4	Liu2	Lin2	zuo4	liang2	mian4	le
		'No, (it's) Liu Lin (that) made the noodle dish.'								

2.2 Contextual felicity study

2.2.1 English

nr	context utterance (canonical sentence)		target utterance
	inducing subject focus	inducing object focus	(canonical sentence)
1	They auctioned off many things today. Peter sold the bicycle.	They auctioned off many things today. John sold the car.	No, John sold the bicycle.
2	The new furniture looks nice. Leni bought the shelves.	The new furniture looks nice. Marlon bought the sofa.	No, Marlon that bought the shelves.
3	The food at the conference was delicious. Milo ate the beef.	The food at the conference was delicious. Tom ate the salad.	No, Tom ate the beef.
4	The boys were really drunk yesterday. Jim drank a lot of vodka.	The boys were really drunk yesterday. Toni drank a lot of beer.	No, Toni drank a lot of vodka.
5	The boys did their chores today. Greg washed the cars.	The boys did their chores today. Mike washed the motorcycles.	No, Mike washed the cars.
6	These women are such wonderful writers. Anna writes poetry.	These women are such wonderful writers. Helen writes novels.	No, Helen writes poetry.
7	Our boys are interested in literature. Andrew reads short stories.	Our boys are interested in literature. Steven reads nonfiction.	No, Steven reads short stories.
8	No one in the office is following the dress code. Marie is wearing a blouse.	No one in the office is following the dress code. Julie is wearing a T-shirt.	No, Julie is wearing a blouse.
9	Some things needed to be repaired in the kitchen. Bob repaired the drainpipe.	Some things needed to be repaired in the kitchen. Glenn repaired the door.	No, Glenn repaired the drainpipe.
10	I bought some new paint for painting the rooms. Mike is painting the walls.	I bought some new paint for painting the rooms. Anton is painting the doors.	No, Anton is painting the walls.
11	The meal was fantastic. Claire cooked the rice.	The meal was fantastic. Jenny cooked the soup.	No, Jenny cooked the rice.
12	The children bought some bread to feed the animals. Tim fed the goats.	The children bought some bread to feed the animals. Ben fed the ducks.	No, Ben fed the goats.
13	The boys caused a lot of damage. Rob smashed the windows.	The boys caused a lot of damage. Finn smashed the china.	No, Finn smashed the windows.
14	The teenagers went to the gym to play some ball. Michael is playing tennis.	The teenagers went to the gym to play some ball. Timo is playing badminton.	No, Timo is playing tennis.
15	The pets were really lovely. Lena stroked the cat.	The pets were really lovely. Maria stroked the dog.	No, Maria stroked the cat.
16	Some of our students were interested in East Asian languages. Paul learned Chinese.	Some of our students were interested in Asian languages. Henry learned Korean.	No, Henry learned Chinese.

2.2.2 German

nr	context utterance (canonical sentence) inducing subject focus	inducing object focus	target utterance (canonical sentence)
1	Endlich weiß ich es: Peter hat das Fahrrad verkauft.	Endlich weiß ich es: Johannes hat das Auto verkauft.	Nein, Johannes hat das Fahrrad verkauft.
2	Die neuen Möbel sehen schick aus. Leni hat das Regal gekauft.	Die neuen Möbel sehen schick aus. Marlon hat das Sofa gekauft.	Nein, Marlon hat das Regal gekauft.
3	Schon komisch, uns war allen schlecht gestern. Milo hat Rind gegessen.	Schon komisch, uns war allen schlecht gestern. Tom hat Salat gegessen.	Nein, Tom hat Rind gegessen.
4	Die Jungs waren ziemlich betrunken gestern. Jens hat viel Wodka getrunken.	Die Jungs waren ziemlich betrunken gestern. Toni hat viel Bier getrunken.	Nein, Toni hat viel Wodka getrunken.
5	In der Halle ist was los. Gregor hat die Autos gewaschen.	In der Halle ist was los. Mike hat die Motorräder gewaschen.	Nein, Mike hat die Autos gewaschen.
6	Die Literatur dieser Frauen ist super! Anna hat die Gedichte geschrieben.	Die Literatur dieser Frauen ist super! Helen hat die Romane geschrieben.	Nein, Helen hat die Gedichte geschrieben.
7	Unsere Jungs haben sich für Literatur interessiert. Andreas hat Kurzgeschichten gelesen.	Unsere Jungs haben sich für Literatur interessiert. Stefan hat Sachbücher gelesen.	Nein, Stefan hat Kurzgeschichten gelesen.
8	Wir hatten eine strenge Kleiderordnung im Büro. Marie hat oft eine Bluse getragen.	Wir hatten eine strenge Kleiderordnung im Büro. Leni hat oft ein T-shirt getragen.	Nein, Leni hat oft eine Bluse getragen.
9	Einige Sachen mussten noch repariert werden. Boris hat den Abfluss repariert.	Einige Sachen mussten noch repariert werden. Josef hat die Küchentür repariert.	Nein, Josef hat den Abfluss repariert.
10	Ich habe damals die Farbe gekauft. Michael hat die Wände gestrichen.	Ich habe damals die Farbe gekauft. Nele hat die Türen gestrichen.	Nein, Nele hat die Wände gestrichen.
11	Das Essen war fantastisch. Clara hat den Reis zubereitet.	Das Essen war fantastisch. Jenny hat die Suppe zubereitet.	Nein, Jenny hat den Reis zubereitet.
12	Im Zoo durften wir die Tiere füttern. Tim hat die Bären gefüttert.	Im Zoo durften wir die Tiere füttern. Lara hat die Enten gefüttert.	Nein, Lara hat die Bären gefüttert.
13	Unsere Gäste haben viel zerstört. Robert hat die Gläser zerschlagen.	Unsere Gäste haben viel zerstört. Lena hat das Porzellan zerschlagen.	Nein, Lena hat die Gläser zerschlagen.
14	Beide mochten sie gern Ballsportarten. Frank hat Tischtennis gespielt.	Beide mochten sie gern Ballsportarten. Timo hat Badminton gespielt.	Nein, Timo hat Tischtennis gespielt.
15	Ihre beiden Haustiere sind so niedlich. Lena hat die Katze gestreichelt.	Ihre beiden Haustiere sind so niedlich. Maria hat den Hund gestreichelt.	Nein, Maria hat die Katze gestreichelt.
16	Sprachen finden die Studenten interessant. Paul hat Chinesisch gelernt.	Sprachen finden die Studenten interessant. Henry hat Koreanisch gelernt.	Nein, Henry hat Chinesisch gelernt.

2.2.3 French

nr	context utterance (canonical sentence) inducing subject focus	inducing object focus	target utterance (canonical sentence)
1	Je le sais enfin: Pierre a vendu le vélo.	Je le sais enfin: Jean a vendu la voiture.	Non, Jean a vendu le vélo.
2	Le nouveau mobilier est élégant. Leila a acheté l'étagère.	Le nouveau mobilier est élégant. Marcel a acheté le canapé.	Non, Marcel a acheté l'étagère.
3	C'est bizarre, nous étions tous mal après la visite au restaurant. Michel a mangé du bœuf.	C'est bizarre, nous étions tous mal après la visite au restaurant. Tom a mangé de la salade.	Non, Tom a mangé du bœuf.
4	Les garçons étaient bien saoul hier. Jules a bu beaucoup de vodka.	Les garçons étaient bien saoul hier. Tony a bu beaucoup de bière.	Non, Tony a bu beaucoup de vodka.
5	Il s'en passe des choses dans le hall. Grégoire a lavé les voitures.	Il s'en passe des choses dans le hall. Martin a lavé les motos.	Non, Martin a lavé les voitures.
6	La littérature de ces femmes est superbe! Anne a écrit les poèmes.	La littérature de ces femmes est superbe! Hélène a écrit les romans.	Non, Hélène a écrit les poèmes.
7	Nos garçons s'intéressaient à la littérature. André a lu des nouvelles.	Nos garçons s'intéressaient à la littérature. Stéphane a lu des ouvrages pratiques.	Non, Stéphane a lu des nouvelles.
8	Il y avait à l'époque un code vestimentaire strict au bureau. Marie portait souvent une blouse.	Il y avait à l'époque un code vestimentaire strict au bureau. Leila portait souvent un T-shirt.	Non, Leila portait souvent une blouse.
9	Certaines choses ont dû être réparées. Brice a réparé l'écoulement.	Certaines choses ont dû être réparées. Joseph a réparé la porte de la cuisine.	Non, Joseph a réparé l'écoulement.
10	J'avais acheté la peinture à l'époque. Mickael a peint les murs.	J'avais acheté la peinture à l'époque. Naël a peint les portes.	Non, Naël a peint les murs.
11	Le repas était fantastique. Clara a préparé le riz.	Le repas était fantastique. Joany a préparé la soupe.	Non, Joany a préparé le riz.
12	Au zoo, nous avons eu le droit de nourrir les animaux. Tristan a nourri les ours.	Au zoo, nous avons eu le droit de nourrir les animaux. Lara a nourri les canards.	Non, Lara a nourri les ours.
13	Nos invités ont cassé beaucoup de choses. Robert a brisé les verres.	Nos invités ont cassé beaucoup de choses. Léa a brisé la porcelaine.	Non, Léa a brisé les verres.
14	Tous les deux aimaient les sports de balle. Franck a joué au tennis.	Tous les deux aimaient les sports de balle. Thibault a joué au badminton.	Non, Thibault a joué au tennis.
15	Ses deux animaux domestiques sont tellement mignons. Léa a caressé le chat.	Ses deux animaux domestiques sont tellement mignons. Mariam a caressé le chien.	Non, Mariam a caressé le chat.
16	Les étudiants trouvent les langues intéressantes. Paul a appris le chinois.	Les étudiants trouvent les langues intéressantes. Henry a appris le coréen.	Non, Jean a vendu le vélo.

2.2.4 Chinese

nr	context (condition canonical)<br>inducing subject focus	inducing object focus	target utterance<br>(canonical sentence)
1	他们聊了车。李伟卖了自行车。	他们聊了车。王南卖了汽车。	不对，王南卖了自行车。
2	新的家具很好看。张乐宁买了书架。	新的家具很好看。马涛买了沙发。	不对，马涛买了书架。
3	在那个酒店吃饭以后， 我们都不舒服了。刘峰吃了牛肉。	在那个酒店吃饭以后， 我们都不舒服了。苏立吃了沙拉。	不对，苏立吃了牛肉。
4	男孩子们昨天真的喝醉了。金欢喝了伏特加。	男孩子们昨天真的喝醉了。郭嘉喝了啤酒。	不对，郭嘉喝了伏特加。
5	大厅里的车看上去不很干净。陈飞洗了汽车。	大厅里的车看上去不很干净。杨海洗了摩托车。	不对，杨海洗了汽车。
6	这些女人写的文学作品很不错。刘艳写了诗。	这些女人写的文学作品很不错。王琳写了小说。	不对，王琳写了诗。
7	男孩对文学很感兴趣。高远喜欢读短篇小说。	男孩对文学很感兴趣。施洋喜欢读纪实文学。	不对，施洋喜欢读短篇小说。
8	办公室里得穿正装。吴芳穿了衬衫。	办公室里得穿正装。周怡穿了T恤衫。	不对，周怡穿了衬衫。
9	厨房里有些东西需要修理了。薛涛修了排水管。	厨房里有些东西需要修理了。章坚修了厨门。	不对，章坚修了排水管。
10	我买了新的油漆粉刷房间。周华粉刷了墙。	我买了新的油漆粉刷房间。姜尚粉刷了门。	不对，姜尚粉刷了墙。
11	这一餐好吃极了。林婷婷煮了米饭。	这一餐好吃极了。徐蕾煮了粥。	不对，徐蕾煮了米饭。
12	他们买了一些面包喂动物吃。李勇喂了羊。	他们买了一些面包喂动物吃。曹立喂了鸭子。	不对，曹立喂了羊。
13	他们造成了很多损失。彭军打碎了窗户。	他们造成了很多损失。何磊打碎了瓷器。	不对，何磊打碎了窗户。
14	他们都去体育馆打球了。唐云在打乒乓球。	他们都去体育馆打球了。冯强在打羽毛球。	不对，冯强在打乒乓球。
15	这些宠物很可爱。朱虹抚摸了猫。	这些宠物很可爱。丁薇抚摸了狗。	不对，丁薇抚摸了猫。
16	我们的一些学生对东亚语言颇有兴趣。钱浩学习中文。	我们的一些学生对东亚语言颇有兴趣。谢南学习韩语。	不对，谢南学习中文。

3 R-code

3.1 Speech production study

```
# LIBRARIES
# data processing
library(reshape2)
library(plyr)
# input/output
library(xlsx)
# mixed models
library(lme4)
library(LMERConvenienceFunctions)
library(lmerTest)
# global settings
setwd(dirname(rstudioapi::getActiveDocumentContext()$path))
# DATA PREPARATION
# read data
speech.dta <- data.frame(read.xlsx(file="speech.dta.xlsx",sheetName = "results"))
speech.dta.m <- melt(speech.dta,
                    id = c("language", "file_name", "participant", "item", "construction",
                    "focus","interval_nr","interval_label", "aoi","T1","T2"),
                    measured = c("fo_1", "fo_2","fo_3","fo_4","fo_5"))
# semitone transformation
speech.dta.m$fo_sem <- 12*log(speech.dta.m$value/50,2)
# data types
speech.dta.m$time <- as.numeric(substring(speech.dta.m$variable,4,4))
speech.dta.m$construction <- factor(speech.dta.m$construction,
                                 levels=c("canonical","cleft"))
speech.dta.m$focus <- factor(speech.dta.m$focus,
                                 levels=c("object","subject"))
```

```
speech.dta.m$participant <- factor(speech.dta.m$participant)

speech.dta.m$item <- factor(speech.dta.m$item)

speech.dta.m$int.time <- paste0(speech.dta.m$interval_nr,"_",speech.dta.m$time)

# language subsets

chi.dta <- droplevels(subset(speech.dta.m,language == "Chinese"))

eng.dta <- droplevels(subset(speech.dta.m,language == "English"))

fre.dta <- droplevels(subset(speech.dta.m,language == "French"))

ger.dta <- droplevels(subset(speech.dta.m,language == "German"))

# subsets for areas of interest

chi.sbj1 <- droplevels(subset(chi.dta,aoi == "subject 1"))

chi.sbj2 <- droplevels(subset(chi.dta,aoi == "subject 2"))

fre.sbj <- droplevels(subset(fre.dta,aoi == "subject"))

eng.sbj <- droplevels(subset(eng.dta,aoi == "subject"))

ger.sbj <- droplevels(subset(ger.dta,aoi == "subject"))

chi.obj1 <- droplevels(subset(chi.dta,aoi == "object 1"))

chi.obj2 <- droplevels(subset(chi.dta,aoi == "object 2"))

fre.obj <- droplevels(subset(fre.dta,aoi == "object"))

eng.obj <- droplevels(subset(eng.dta,aoi == "object"))

ger.obj <- droplevels(subset(ger.dta,aoi == "object"))

# LINEAR MIXED-EFFECTS MODELS

# Parsimonious random-effects structure

# testing whether a random-effects converges in all steps of the step-algorithm

# for backwards model selection

# random.0 (=maximal RE structure): does not converge in all languages, e.g., not in
Chinese (subject/object AOI).

random.0 <- function(dta){

  lang.lmer <- lmer(fo_sem ~ construction*focus*time +

                    (1 + construction + focus | participant) +

                    (1 + construction + focus | item),

                  data=dta, REML=FALSE)
```

Correction by Focus

```
  s <<- step(lang.lmer,reduce.fixed = T, reduce.random = F)
  print(s)
  winner <<-get_model(s)
}
random.0(chi.sbj1)#failed to converge
random.0(chi.obj1)#failed to converge
```

```
# Start with reducing the slopes with items (since this random effect has less df)
# random.11 does not converge in all languages, e.g., Chinese subject/object AOI
```

```
random.11 <- function(dta){
  lang.lmer <- lmer(fo_sem ~ construction*focus*time +
                      (1 + focus + construction | participant) +
                      (1 + focus  | item),
                    data=dta, REML=FALSE)
  s <<- step(lang.lmer,reduce.fixed = T, reduce.random = F)
  print(s)
  winner <<-get_model(s)
}
random.11(chi.sbj1)#failed to converge
random.11(chi.obj1)#failed to converge
```

```
# random.12 does not converge in all languages, e.g., Chinese subject/object AOI
```

```
random.12 <- function(dta){
  lang.lmer <- lmer(fo_sem ~ construction*focus*time +
                      (1 + focus + construction | participant) +
                      (1 + construction  | item),
                    data=dta, REML=FALSE)
  s <<- step(lang.lmer,reduce.fixed = T, reduce.random = F)
  print(s)
  winner <<- get_model(s)
}
```

```
random.12(chi.sbj1)#failed to converge
random.12(chi.obj1)#failed to converge
```

```
# random.13 does not converge in the subject AOI analyses of all languages, e.g., English
```

```
random.13 <- function(dta){
  lang.lmer <- lmer(fo_sem ~ construction*focus*time +
                      (1 + focus + construction | participant) +
                      (1 | item),
                    data=dta, REML=FALSE)
  s <<- step(lang.lmer,reduce.fixed = T, reduce.random = F)
  print(s)
  winner <<- get_model(s)
}
random.13(eng.sbj)#failed to converge
```

```
# random.13 converges in the object AOI analyses of all languages
```

```
random.13(chi.obj1)#converges
  chi.obj1.win <- winner
random.13(chi.obj2)#converges
  chi.obj2.win <- winner
random.13(fre.obj)#converges
  fre.obj.win <- winner
random.13(eng.obj)#converges
  eng.obj.win <- winner
random.13(ger.obj)#converges
  ger.obj.win <- winner
```

```
# random.21 converges in the subject AOI analyses of all languages
```

```
random.21 <- function(dta){
  lang.lmer <- lmer(fo_sem ~ construction*focus*time +
                      (1 + construction | participant) +
                      (1 | item),
```

```
                      data=dta, REML=FALSE)
  s <<- step(lang.lmer,reduce.fixed = T, reduce.random = F)
  print(s)
  winner <<- get_model(s)
}
random.21(chi.sbj1)#converges
  chi.sbj1.win <- winner
random.21(chi.sbj2)#converges
  chi.sbj2.win <- winner
random.21(fre.sbj)#converges
  fre.sbj.win <- winner
random.21(eng.sbj)#converges
  eng.sbj.win <- winner
random.21(ger.sbj)#converges
  ger.sbj.win <- winner
# MODEL COMPARISON
# subject AOI
chi.sbj1.win1 = update(chi.sbj1.win, . ~ . - construction:time)
chi.sbj1.win2 = update(chi.sbj1.win, . ~ . - focus:time)
  anova(chi.sbj1.win,chi.sbj1.win1)
  anova(chi.sbj1.win,chi.sbj1.win2)
chi.sbj2.win1 = update(chi.sbj2.win, . ~ . - focus:time)
  anova(chi.sbj2.win,chi.sbj2.win1)
fre.sbj.win1 = update(fre.sbj.win, . ~ . - construction:focus)
fre.sbj.win2 = update(fre.sbj.win, . ~ . - focus:time)
  anova(fre.sbj.win,fre.sbj.win1)
  anova(fre.sbj.win,fre.sbj.win2)
eng.sbj.win1 = update(eng.sbj.win, . ~ . - construction:focus)
eng.sbj.win2 = update(eng.sbj.win, . ~ . - construction:time)
eng.sbj.win3 = update(eng.sbj.win, . ~ . - focus:time)
```

```
  anova(eng.sbj.win,eng.sbj.win1)
  anova(eng.sbj.win,eng.sbj.win2)
  anova(eng.sbj.win,eng.sbj.win3)
ger.sbj.win1 = update(ger.sbj.win, . ~ . - construction:focus:time)
  anova(ger.sbj.win,ger.sbj.win1)
# object AOI
chi.obj1.win1 = update(chi.obj1.win, . ~ . - focus:time)
  anova(chi.obj1.win,chi.obj1.win1)
chi.obj2.win1 = update(chi.obj2.win, . ~ . - focus:time)
chi.obj2.win2 = update(chi.obj2.win, . ~ . - construction)
  anova(chi.obj2.win,chi.obj2.win1)
  anova(chi.obj2.win,chi.obj2.win2)
eng.obj.win1 = update(eng.obj.win, . ~ . - focus:time)
  anova(eng.obj.win,eng.obj.win1)
fre.obj.win1 = update(fre.obj.win, . ~ . - construction:focus)
fre.obj.win2 = update(fre.obj.win, . ~ . - focus:time)
  anova(fre.obj.win,fre.obj.win1)
  anova(fre.obj.win,fre.obj.win2)
ger.obj.win1 = update(ger.obj.win, . ~ . - focus:time)
  anova(ger.obj.win,ger.obj.win1)
```

3.2 Contextual felicity study

```
# LIBRARIES
# mixed models
library(ordinal)
# data processing
library(xlsx)
library(plyr)
# global settings
setwd(dirname(rstudioapi::getActiveDocumentContext()$path))
```

Correction by Focus

```
# DATA PREPARATION

# read data

scores <- data.frame(read.xlsx(file="felicity.dta.xlsx",sheetName = "results"))

# data types

scores$participant <- as.factor(scores$participant)

scores$item <- as.factor(scores$item)

scores$construction <- factor(scores$construction,levels=c("canonical","cleft"))

scores$focus <- factor(scores$focus,levels=c("object","subject"))

scores$context <- factor(scores$context,levels=c("canonical","cleft"))

scores$rating <- as.numeric(scores$rating)

# Creating language subsets

chi.scores <- droplevels(subset(scores,language == "Chinese"))

eng.scores <- droplevels(subset(scores,language == "English"))

fre.scores <- droplevels(subset(scores,language == "French"))

ger.scores <- droplevels(subset(scores,language == "German"))

# ORDINAL REGRESSION MODELS

# Control settings

# useMatrix = T is recommended for models with more than one random effects

# see https://www.rdocumentation.org/packages/ordinal/versions/2019.12-
10/topics/clmm.control

# ratings as ordinal data

chi.scores$rating <- factor(chi.scores$rating, ordered=TRUE)

fre.scores$rating <- factor(fre.scores$rating, ordered=TRUE)

eng.scores$rating <- factor(eng.scores$rating, ordered=TRUE)

ger.scores$rating <- factor(ger.scores$rating, ordered=TRUE)


# Maximal fixed-effects model with the most parsimonious RE structure

chi.0 = clmm(rating ~ construction * focus * context +

               (1+ construction + focus + context|participant) +

               (1+ construction + focus + context|item),
```

```
               data=chi.scores, control = clmm.control(useMatrix = T))
fre.0 = clmm(rating ~ construction * focus * context +
               (1+ construction + focus + context|participant) +
               (1+ construction + focus + context|item),
               data=fre.scores, control = clmm.control(useMatrix = T))
eng.0 = clmm(rating ~ construction * focus * context +
               (1+ construction + focus + context|participant) +
               (1+ construction + focus + context|item),
               data=eng.scores, control = clmm.control(useMatrix = T))
ger.0 = clmm(rating ~ construction * focus * context +
               (1+ construction + focus + context|participant) +
               (1+ construction + focus + context|item),
               data=ger.scores, control = clmm.control(useMatrix = T))
```

```
# MODEL COMPARISON
# The comments summarize the result:
# "n.s." = not significant; "*","**","***": significance levels
# Chinese
```

```
chi.1 = update(chi.0,. ~ . - construction : focus : context)
  anova(chi.0,chi.1)# n.s.
chi.11 = update(chi.1,. ~ . - construction : focus )
  anova(chi.1,chi.11)# *
chi.12 = update(chi.1,. ~ . - construction : context)
  anova(chi.1,chi.12)# *
chi.13 = update(chi.1,. ~ . - focus : context)
  anova(chi.1,chi.13)# n.s.
chi.131 = update(chi.13,. ~ . - construction : focus)
  anova(chi.13,chi.131)# ***
chi.132 = update(chi.13,. ~ . - construction : context)
  anova(chi.13,chi.132)# *
```

```
chi.winner <- chi.13
  summary(chi.winner)
# French
fre.1 = update(fre.0,. ~ . - construction : focus : context)
  anova(fre.0,fre.1)# n.s.
fre.11 = update(fre.1,. ~ . - construction : focus)# failed to converge
  anova(fre.1,fre.11)# ***
fre.12 = update(fre.1,. ~ . - construction : context)
  anova(fre.1,fre.12)# n.s.
fre.13 = update(fre.1,. ~ . - focus : context)
  anova(fre.1,fre.13)# *
fre.121 = update(fre.12,. ~ . - focus : context)
  anova(fre.12,fre.121)# *
fre.122 = update(fre.12,. ~ . - construction : focus)
  anova(fre.12,fre.122)# ***
fre.winner <- fre.12
  summary(fre.winner)
# English
eng.1 = update(eng.0,. ~ . - construction : focus : context)
  anova(eng.0,eng.1)# **
eng.winner <- eng.0
  summary(eng.winner)
# German
ger.1 = update(ger.0,. ~ . - construction : focus : context)
  anova(ger.0,ger.1)# *
ger.winner <- ger.0
  summary(ger.winner)
```

4 Ordinal regression: threshold intercepts

language	1|2	2|3	3|4	4|5	5|6	6|7
English	–7.770	–5.662	–4.718	–3.717	–2.478	–0.836
German	–5.726	–4.448	–3.530	–2.955	–2.014	–0.498
French	–6.413	–5.115	–4.054	–3.365	–2.443	–0.662
Chinese	–4.797	–3.841	–3.032	–2.508	–1.710	–0.042
average	–6.177	–4.767	–3.834	–3.136	–2.161	–0.510
